# Focusing on Prostate-Specific Membrane Antigen in Precision Diagnosis and Treatment of Prostate Cancer

**DOI:** 10.3390/biomedicines14020482

**Published:** 2026-02-22

**Authors:** Xinyi Ren, Lingling Zhang, Ran An, Hongchen Song, Mingjun Shi, Zhenchang Wang

**Affiliations:** 1 Department of Radiology, Beijing Friendship Hospital, Capital Medical University, Beijing 100050, China; renxinyi96@163.com (X.R.); anran8051@163.com (R.A.); 2 Department of Radiology, Beijing Tiantan Hospital, Capital Medical University, Beijing 100070, China; zhanglingling_mail@163.com; 3 Department of Surgery, Hebei Medical University, Shijiazhuang 050000, China; songhcupup@163.com; 4 Department of Urology, Beijing Friendship Hospital, Capital Medical University, Beijing 100050, China; 5 Institute of Urology, Beijing Municipal Health Commission, Beijing 100054, China

**Keywords:** PSMA, prostate cancer, targeted imaging, targeted therapy

## Abstract

Prostate cancer (PCa) is the most common malignant tumor of the male genitourinary system, and its incidence and mortality have shown a marked global increase in recent years. Prostate-specific membrane antigen (PSMA), a type II transmembrane glycoprotein highly expressed in PCa cells, has emerged as a vital molecular target in the field of PCa precision diagnosis and therapy. In recent years, significant advances have been achieved in PSMA-based molecular imaging, radioligand therapy, and the development of novel targeted drugs. This review aims to summarize and critically discuss recent advances in PSMA-targeted molecular imaging, radioligand therapy, and emerging therapeutic strategies, highlighting their roles in precision diagnosis and personalized treatment of PCa. PSMA positron emission tomography/computed tomography (PET/CT) imaging using radionuclides such as ^68^Ga and ^18^F has markedly improved the accuracy of primary tumor staging, localization of recurrent lesions, and therapeutic response assessment. Radioligand therapies, such as ^177^Lu-PSMA-617 and ^225^Ac-PSMA-617, have prolonged survival and demonstrated symptomatic benefits in multiple clinical trials, and are now applied in early disease stages, including chemotherapy-naïve and hormone-sensitive settings. Meanwhile, PSMA-targeted antibodies and antibody–drug conjugates (PSMA-ADCs), as well as bispecific T-cell engagers (BiTEs) and chimeric antigen receptor T-cell (CAR-T) therapies, are constantly being optimized and show promising clinical potential. Furthermore, PSMA-targeted nanoplatforms enable precise delivery of chemotherapeutic agents, photosensitizers, or imaging probes, achieving integrated diagnosis and therapy with multimodal imaging guidance, and offering new strategies for individualized treatment. Taken together, the evidence summarized in this review highlights PSMA as a pivotal molecular target supporting precision diagnosis and personalized treatment across the continuum of prostate cancer management.

## 1. Introduction

Cancer remains a leading cause of morbidity and mortality worldwide and continues to represent a major global health burden [1]. Prostate cancer (PCa) is one of the most common malignant tumors in men worldwide, with approximately 1.5 million new cases and nearly 400,000 deaths reported in 2022 [2]. With an aging global population and increasing life expectancy, the burden of PCa is expected to increase. In 2024, the Lancet Commission reported that without additional interventions, the number of new PCa cases is projected to increase from 1.4 million in 2020 to 2.9 million in 2040—an approximately 107% rise [3]. Currently, established risk factors include age, family history, certain gene mutations (such as *BRCA1* and *BRCA2*), and hereditary conditions (such as Lynch syndrome). Beyond these well-established genetic and hormonal factors, emerging in silico evidence suggests that chronic bacterial infections may theoretically contribute to prostate cancer-related cellular dysregulation through pathogen protein targeting of host subcellular compartments [4,5,6]. Among these, factors such as the overexpression of prostate-specific membrane antigen (PSMA) and *BRCA2* gene mutations are both indicative of a more aggressive phenotypic characteristic [7].

Although significant progress has been made in precision diagnosis and treatment of PCa, the accuracy of diagnosis and the individualization of therapy remain unsatisfactory. Despite the wide use of prostate-specific antigen (PSA) and PSA density (PSAD) for PCa screening, their diagnostic specificity is limited. Moreover, PSA levels within the “gray zone” of 4–10 ng/mL further increase diagnostic uncertainty, often leading to overdiagnosis and unnecessary biopsies [8]. The combination of multiparametric magnetic resonance imaging (mpMRI) and PSA testing has significantly improved the detection rate of PCa and, to some extent, reduced overdiagnosis and overtreatment [9]. However, its diagnostic performance remains limited in heterogeneous patient populations with Prostate Imaging Reporting and Data System (PI-RADS) scores of 3 or 4 [10]. In fact, invasive prostate biopsy remains the gold standard for PCa diagnosis, although it may overdiagnose non-clinically significant PCa (non-csPCa) and cause procedure-related complications such as infection and urinary retention. The therapeutic landscape for PCa is relatively well-established and encompasses a combination of approaches, including active surveillance, radical surgery, radiotherapy, androgen deprivation therapy (ADT), chemotherapy, and emerging targeted therapies. Systemic treatment based on androgen deprivation remains the mainstay for patients with locally advanced or metastatic disease, and the introduction of novel hormonal agents (such as abiraterone and apalutamide) and targeted drugs (such as poly (ADP-ribose) polymerase (PARP) inhibitors) has significantly prolonged survival. However, almost all patients eventually progress to castration-resistant prostate cancer (CRPC), characterized by marked tumor heterogeneity, limited subsequent treatment options, and the emergence of drug resistance. Therefore, the development of precise and effective novel therapeutic strategies remains a key challenge for future research.

PSMA, encoded by the *FOLH1* gene, is a type II transmembrane glycoprotein composed of 750 amino acid residues, with an extracellular domain comprising approximately 704 amino acids. Under physiological conditions, PSMA is expressed at low levels in the small intestine, proximal renal tubules, salivary glands, and prostatic epithelium [11]. In tumors, PSMA is expressed in the neovasculature of various malignancies; however, its expression is markedly upregulated in both primary and metastatic lesions of PCa and is further elevated in most CRPC cases [12,13]. Moreover, high PSMA expression is positively correlated with a higher Gleason grade and poor prognosis [14]. For example, the 5-year recurrence-free survival rate after radical prostatectomy is significantly negatively correlated with PSMA expression level, and patients with high PSMA expression have a risk of recurrence more than 4-fold higher than that in controls [15]. Several studies have demonstrated that PSMA expression is associated with the molecular subtypes of metastatic PCa [16,17]. The androgen receptor (AR)-positive subtype generally has high PSMA expression, whereas the AR-negative/neuroendocrine (NE)-positive neuroendocrine subtype often shows low or no PSMA expression (accounting for approximately 15% of cases with CRPC), which partly explains the molecular heterogeneity observed in advanced tumors. The efficacy of PSMA in managing PCa has been demonstrated in clinical settings, with comprehensive coverage from precise diagnosis to targeted therapy. In recent years, significant breakthroughs have been made in PSMA-based noninvasive molecular detection, advanced imaging technologies (such as JVZ-007), and targeted therapies (such as nanoplatform). Given the pivotal role of PSMA in PCa, this review summarizes recent advances in PSMA-targeted agents for diagnosis and therapy, including PET imaging, antibody-based approaches, and nanomaterials, among others, highlighting their clinical applications and translational potential.

## 2. Application of PSMA in PCa Diagnosis

Accurate diagnosis and staging remain critical challenges in PCa management, particularly in the detection of clinically significant disease and metastatic involvement. Conventional diagnostic approaches, including serum PSA testing and anatomical imaging (CT/MRI), are limited by suboptimal specificity and sensitivity, especially for small-volume lesions and lymph node metastases. These unmet diagnostic needs have driven the development of PSMA-targeted molecular imaging and diagnostic strategies.

### 2.1. Noninvasive Liquid Biopsy Based on PSMA

Noninvasive detection of PCa through exosomes or extracellular vesicles (EVs), circulating tumor cells (CTCs), and circulating tumor DNA (ctDNA) in blood or urine is an emerging field. ctDNA analysis enables identification of key genomic alterations for molecular stratification [18], while CTC- and EV-based assays demonstrate improved diagnostic accuracy over PSA testing and may substantially reduce unnecessary biopsy [19,20].

PSMA has also emerged in the field of noninvasive PCa detection, demonstrating good diagnostic potential and value for dynamic efficacy monitoring [21]. Wang et al. [22] found that the PSMA levels in urine-derived exosomes differed significantly between patients with PCa and benign prostatic hyperplasia. The diagnostic area under the curve (AUC) for PCa and csPCa reached 0.88 [95% confidence interval (CI) 0.83–0.93] and 0.83 [95% CI 0.76–0.89], respectively—substantially outperforming conventional PSA testing—and could reduce unnecessary biopsies by 41% while missing only 0.7% of csPCa cases. Matijašević Joković et al. [23] also indicated that the expression level of exosomal PSMA in the plasma of patients with PCa was significantly higher than that of patients with benign prostatic hyperplasia, suggesting the potential for noninvasive screening and diagnosis of PCa. Gupta et al. [24] developed a liquid biopsy method capable of detecting PSMA expression in CTCs at the single-cell level and assessing tumor heterogeneity. This method not only guides patient selection for PSMA-targeted therapies but also holds potential prognostic and pharmacodynamic predictive value.

Overall, the current applications of PSMA in liquid biopsy are evolving from basic biomarker exploration to clinical implementation. These include PSMA detection in urine- or blood-derived exosomes, identification of PSMA-positive CTCs, and ctDNA-based precision expression profiling, which offer innovative tools for early diagnosis and therapeutic response monitoring. However, these studies are still at the preliminary validation stage. Their clinical value should be confirmed by large-scale population studies and prospective research in the future [25].

### 2.2. Application of PSMA in the Diagnosis of Primary PCa

**Figure 1:**
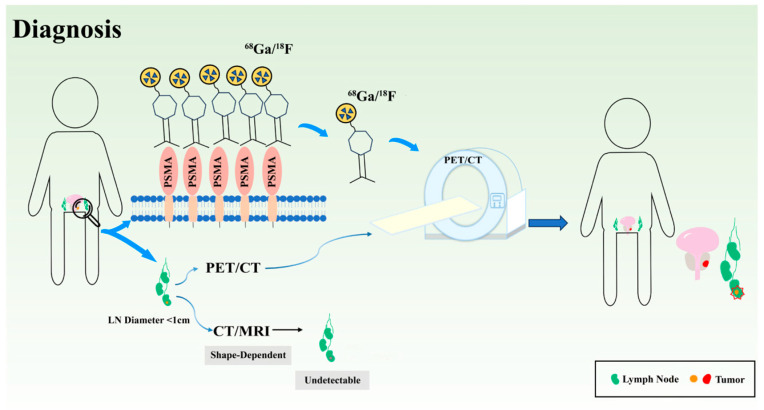
PSMA-targeted PET/CT in PCa and lymph node metastasis. PET/CT, positron emission tomography/computed tomography; LN, lymph node.

Novel imaging technologies such as PSMA positron emission tomography/computed tomography (PET/CT) demonstrate significantly higher diagnostic accuracy than conventional imaging modalities (such as standard PET/CT, MRI, and bone scans), enabling more precise detection of minute metastases and facilitating accurate staging and personalized treatment plans [9,26,27]. Multiple PSMA-targeted antibodies and small-molecule tracers have been developed recently and are being optimized for the accurate diagnosis of PCa (Figure 1).

#### 2.2.1. Modification and Optimization of Anti-PSMA Antibodies

The first PSMA-targeted radio-immunoscintigraphic agent approved for PCa, ProstaScint (Capromab Pendetide), utilizes a murine monoclonal antibody, 7E11, as the targeting vector, conjugated with the radionuclide ^111^In, to visualize positive lesions through single-photon computed tomography (SPECT) imaging. In the initial staging, its performance was superior to that of conventional imaging, with a sensitivity of 52–62% and a specificity of 72–96% [28]. However, the murine antibody can trigger a Human Anti-Mouse Antibody (HAMA) response in the human body, which affects the efficacy of repeated use and can potentially interfere with other blood tests. Moreover, because 7E11 exclusively binds to the intracellular domain of PSMA, within cells undergoing necrosis/apoptosis or those with compromised membrane integrity, its capacity to detect the abundant and clinically significant population of viable tumor cells with intact membranes is severely limited; these limitations have driven the development of superior tracers targeting the extracellular domain of PSMA, thereby demonstrating the decisive impact of “target accessibility” on the diagnostic performance of molecular imaging technology.

J591, developed via continuous improvements, is the first humanized antibody to successfully recognize the extracellular domain of PSMA, significantly overcoming the limitations of 7E11. When labeled with ^89^Zr, J591 enabled the specific visualization of PCa lesions in a nude mouse xenograft model (LNCaP as PSMA-positive and PC-3 as the negative control). The results showed that ^89^Zr-DFO-J591 uptake in LNCaP-derived tumors increased over time, reaching approximately 45% ID/g at 144 h, whereas uptake in PSMA-negative PC-3-derived tumors remained minimal, indicating excellent target specificity. Moreover, the tumor-to-muscle ratio exceeded 20 between 48 h and 144 h, highlighting its outstanding imaging contrast [29].

Considering that the large molecular weight of full-length PSMA monoclonal antibodies negatively affects imaging quality, the development of small-molecule PSMA antibodies has become a research focus. IAB2M is a genetically engineered mini-body that retains the PSMA-binding variable region of J591 while removing its Fc fragment, thereby reducing its molecular weight from 150 kDa to 80 kDa [30]. Pandit-Taskar et al. [30] conducted the first-in-human study on ^89^Zr-Df-IAB2M, enrolling 18 patients with metastatic PCa, to systematically evaluate its pharmacokinetics, tissue biodistribution, and dosimetry characteristics. The results showed that ^89^Zr-Df-IAB2M had good safety and tolerability, with a markedly shorter optimal imaging time than the full-length J591 antibody. Dose estimation indicated that the absorbed doses for the liver, kidneys, and bone marrow were 1.67, 1.36, and 0.32 mGy/MBq, respectively, with an effective whole-body dose of 0.41 mSv/MBq. All the estimated doses were within acceptable limits (NCT01923727).

Nanobodies (Nbs) represent a further improvement of monoclonal antibodies. They are antibody-derived fragments (typically the variable domain of a heavy-chain antibody) with a size of approximately 15 kDa. They largely retain the ability to bind to specific antigens, offering faster pharmacokinetics and lower immunogenicity [31]. Researchers have successfully developed imaging probes by labeling PSMA-targeted nanobodies with radionuclides or contrast agents. For example, one study reported a ^111^In-labeled anti-PSMA nanobody (JVZ-007) and systematically evaluated its SPECT/CT imaging performance in a PCa mouse model [32]. The investigators screened for JVZ-007 using alpacas’ immunization and phage display. A single cysteine residue was introduced at the C-terminus to enable site-specific radiolabeling, and maleimide diethylene-triamine-pentaacetate (DTPA) was used to chelate ^111^In while preserving its antigen-binding activity. In vitro experiments confirmed that JVZ-007 exhibits specific binding and internalization in PSMA-positive cell lines. In a PC-310 (PSMA-positive) mouse xenograft model, clear SPECT/CT visualization was achieved as early as 3 h after injection, whereas PSMA-negative PC-3 tumors showed almost no tracer accumulation. Furthermore, structural optimization combined with co-injection of gelofusine and lysine reduced renal uptake in mice by approximately 10-fold, effectively overcoming high kidney retention and improving safety. Fan et al. [33] developed a PSMA-targeted ultrasound nanobubble containing an anti-PSMA nanobody with an average diameter of 487.60 ± 33.55 nm. This size allows it to penetrate the vascular barrier of the tumor. In vitro experiments confirmed that nanobody conjugation confers high specificity, allowing nanobubbles to significantly bind to PSMA-positive PCa cells. In a mouse xenograft model (PSMA-positive), in vivo ultrasound imaging demonstrated that, compared with unmodified control nanobubbles, the PSMA-targeted nanobubbles produced a higher peak intensity (21.47 ± 0.60 vs. 19.62 ± 0.44, *p* < 0.05) and longer enhancement duration (22.58 ± 0.74 vs. 20.20 ± 0.28, *p* < 0.05) at PSMA-positive tumor sites, underscoring their potential to enhance the accuracy and sensitivity of ultrasound-based PCa diagnosis.

#### 2.2.2. Advances and Optimization of PSMA Chelated Tracers

^68^Ga-PSMA-11, which uses HBED-CC as the chelating agent, was the first PET/CT imaging tracer approved by the Food and Drug Administration for PCa. Emmett et al. [34] were the first to propose the PRIMARY Score, which is based on ^68^Ga-PSMA-11 PET/CT. This study included 291 biopsy-naïve patients who previously underwent mpMRI, with tissue biopsy serving as the gold standard for all cases. Based on the pattern and intensity of intra-prostatic PSMA uptake, PET images were classified into five categories: scores of 1–2 were defined as low risk and scores of 3–5 as high risk. The results showed that high PRIMARY Scores (3–5) were strongly associated with csPCa (International Society of Urological Pathology Grade ≥ 2), with a sensitivity of 88% and a negative predictive value (NPV) of 81%, both outperforming mpMRI alone (sensitivity ~83%, NPV 72%). Notably, patients with a score of 5 had a 100% detection rate for csPCa. This study suggests that the ^68^Ga-PSMA-11 PRIMARY Score can serve as a reliable risk stratification tool to improve the diagnostic accuracy of csPCa, complement the limitations of mpMRI, and provide guidance for biopsy target selection while reducing unnecessary biopsies. In a prospective study, Sonni et al. [35] found that ^68^Ga-PSMA-11 PET/CT and mpMRI had comparable accuracies for intraglandular tumor localization. However, mpMRI was superior to PSMA PET/CT in identifying extra-prostatic extensions (EPEs) (AUC 0.79 vs. 0.59, *p* = 0.002) (NCT03368547).

PSMA-I&T uses DOTA as a chelating agent, giving it a broad application range. It can be combined with ^68^Ga for diagnosis and ^177^Lu for therapeutic purposes. Traditional PET/CT relies on images obtained at a single time point for disease diagnosis. However, a multi-timepoint ^68^Ga-PSMA I&T PET/CT study by Schmuck et al. [36] demonstrated that both early dynamic imaging and delayed imaging can effectively identify tumor lesions. When the imaging time was delayed up to 180 min postinjection, the tumor-to-background contrast improved significantly, enhancing lesion visualization and delineation. Furthermore, the peak of standardized uptake value (SUV_peak_) at the delayed time point showed a positive correlation with the Gleason score, suggesting that multi-time point PSMA PET/CT can not only enhance lesion visualization but may also reflect the biological aggressiveness of the tumor. Cytawa et al. [37] reported that SUV_max_ was positively correlated with the PSA level of patients and Gleason score. McCarthy et al. [38] compared the similarities and differences between ^68^Ga-PSMA-11 (^68^Ga HBED-PSMA) and ^68^Ga-PSMA-I&T in PCa imaging diagnosis by enrolling 19 patients who underwent PET/CT with both tracers within 2 weeks. A total of 47 suspected PCa lesions (including bone, lymph node, and intra-prostatic lesions) were identified. Except for two lymph nodes smaller than 4 mm, all lesions were detected by both tracers, yielding a concordance rate of 96%. In terms of semi-quantitative parameters, PSMA-11 demonstrated significantly higher SUV_peak_ values within lesions than PSMA-I&T (*p* < 0.001), indicating a slightly superior tracer–target binding efficiency. Conversely, PSMA-I&T exhibited significantly higher SUV_mean_ values in the left ventricular blood pool and bone marrow background than PSMA-11 (blood pool *p* < 0.001; bone marrow *p* < 0.05), suggesting more pronounced non-specific background activity. Therefore, although both tracers showed comparable lesion detection performance in most cases, PSMA-11 provides better image contrast and micro-lesion visualization. However, as PSMA-11 cannot form sufficiently stable complexes with ^177^Lu for in vivo use, its diagnostic applications are limited. These findings show that each tracer has unique advantages.

PCa has a relatively low glycolytic rate, and ^18^F-FDG performs poorly in diagnostic assessments. ^18^F-DCFBC was one of the first ^18^F-labeled small-molecule tracers targeting PSMA for clinical evaluation, and its use in primary PCa staging laid the groundwork for the development of second-generation PSMA tracers. Rowe et al. [39] and Turkbey et al. [40] assessed the role of ^18^F-DCFBC PET/CT in detecting and characterizing primary PCa and compared its diagnostic performance with that of MRI (NCT01496157, NCT02190279). Both studies showed that although ^18^F-DCFBC PET/CT was less sensitive than MRI in identifying malignant lesions, it had higher specificity (up to 96%) and could distinguish malignant tumors from benign prostatic hyperplasia. Moreover, Rowe et al. [39] found that ^18^F-DCFBC PET/CT could more specifically detect clinically significant, high-grade, and large-volume tumors (e.g., Gleason scores 8 and 9). They also concluded that the tumor uptake of ^18^F-DCFBC was positively correlated with tumor Gleason score, PSMA expression, and PSA levels.

Compared with ^18^F-DCFBC, ^18^F-DCFPyL has significantly improved affinity, pharmacokinetics, and imaging contrast, resulting in better diagnostic performance in primary PCa staging. ^18^F-DCFPyL is a urea-based PSMA inhibitor characterized by higher binding affinity (Kd ≈ 1.1 nM) and lower non-specific blood-pool background [41]. Zhou et al. [42] systematically evaluated the diagnostic value of ^18^F-DCFPyL PET/CT in newly diagnosed patients with PCa and its role in risk stratification. This study included 62 patients with untreated PCa cancer. The relationship between these parameters and D’ Amico risk stratification was analyzed by measuring primary lesion SUV_max_, SUV_mean_, total lesion PSMA uptake (TL-PSMA), tumor volume (PSMA-TV), and prostate/muscle ratio (P/M ratio). The results showed that patients in the high-risk group had significantly higher SUV_max_, SUV_mean_, TL-PSMA, and P/M ratios than those in the low- and intermediate-risk groups (*p* < 0.05). Among these parameters, the P/M ratio demonstrated the best discriminative ability in distinguishing between different risk groups, with a c-statistic of 0.97 for differentiating between the low- and high-risk groups. The P/M ratio is also positively correlated with PSA levels, suggesting that it reflects biological tumor activity and disease aggressiveness. Therefore, ^18^F-DCFPyL PET/CT can not only accurately locate the primary lesion but also assist in risk assessment through quantitative parameters, providing a potential imaging basis for early stratification and individualized treatment decisions for high-risk patients.

The standout features of ^18^F-PSMA-1007 include hepatobiliary metabolism and minimal urinary excretion. On average, only 1.2% of the injected activity is excreted in the urine within 0–2 h after injection [43]. This effectively solves the problem of difficult imaging of local pelvic lesions caused by high background radioactivity in the bladder, which is common with other PSMA tracers. Ye et al. [44] compared the diagnostic value of ^18^F-PSMA-1007 PET/CT and MRI in patients with suspected PCa and found that PET/CT had a sensitivity and accuracy of 95.1%, significantly surpassing MRI (82.9%), highlighting PET scan’s unique advantage in locating the primary PCa lesion. In local staging, a prospective matched-pair study (NCT05141760) [45] compared the staging accuracy of ^18^F-PSMA-1007 PET/CT with mpMRI in intermediate- to high-risk patients scheduled for radical prostatectomy. Among 134 patients, ^18^F-PSMA-1007 PET/CT correctly identified the pathological stage in 61 (45%) cases, compared with 38 (28%) for mpMRI (*p* = 0.003). Furthermore, PET/CT outperformed mpMRI in detecting EPE (*p* < 0.05). Both Pattison et al. and Rauscher et al. [46,47] compared the imaging performance of ^68^Ga-PSMA-11 and ^18^F-PSMA-1007. The former included patients with newly diagnosed disease, biochemical recurrence (BCR), or metastatic restaging, whereas the latter focused exclusively on BCR. Results showed a high degree of concordance between ^68^Ga-PSMA-11 and ^18^F-PSMA-1007 in overall American Joint Committee on Cancer (AJCC) prognostic staging (both 92%) and the number of lesions detected (126 vs. 124). However, because ^18^F-PSMA-1007 is primarily cleared through the hepatobiliary route, it shows markedly reduced bladder activity, facilitating the evaluation of the prostatic bed and pelvic lymph nodes. Both studies also pointed out that ^18^F-PSMA-1007 has a higher false-positive rate, particularly in areas of physiological uptake, such as the ganglia, bones, and liver. Rauscher et al. [47] also reported that the number of benign lesions detected using ^18^F-PSMA-1007 was approximately five times that detected using ^68^Ga-PSMA-11. Therefore, although ^18^F-PSMA-1007 offers certain advantages, image interpretation must be combined with additional clinical information to achieve accurate differentiation.

#### 2.2.3. The Value of PSMA in Prostate Biopsy

Prostate biopsy is the mainstay of PCa diagnosis and is the key to detecting clinically significant PCa. Despite the successful implementation of mpMRI for prostate diseases, selecting appropriate patients for biopsy remains difficult, particularly when dealing with heterogeneous subgroups. Although the MRI–ultrasound software-based fusion method is becoming popular and thought to significantly improve biopsy precision, it is still not routinely used in most centers.

In recent years, several prospective studies have directly compared PSMA-PET-guided biopsy (PSMA-TB) with MRI-guided biopsy (MRI-TB) in PCa diagnosis. Two consecutive prospective controlled trials conducted by Pepe et al. in 2022 and 2023 showed that the detection rates of csPCa using PSMA-TB and MRI-TB were similar, suggesting that they could be interchangeable or complementary [48,49]. In a real-world, multicenter cohort study, Checcucci et al. [50] reported that the detection rate of PSMA-TB for csPCa was superior to that of standard systematic biopsy (0.36 ± 0.44 vs. 0.21 ± 0.30, *p* < 0.05) but comparable to that of MRI-TB (0.36 ± 0.44 vs. 0.47 ± 0.34). The study also indicated that csPCa was more easily detected when the lesion’s SUV_max_ was ≥4.8, suggesting that molecular imaging parameters can serve as a basis for targeted biopsy. Furthermore, in a specific high-risk subgroup of patients with negative MRI results, Bianchi et al. [51] showed that PSMA-TB could significantly increase the detection rate of csPCa, providing an effective supplement. Notably, Chow et al. [52] demonstrated that combining PSMA-TB with mpMRI interpretation enhanced diagnostic performance at both patient and lesion levels, increasing sensitivity and NPV compared with mpMRI alone (*p* ≤ 0.05). This finding strongly supports avoiding unnecessary biopsies in low-risk patients.

In summary, PSMA PET/CT is valuable in the detection of primary PCa, particularly in MRI-negative or equivocal cases. PSMA-guided biopsy strategies can potentially reduce unnecessary procedures while improving the detection rate of clinically significant diseases. However, the current evidence is limited by small sample sizes, differences in radiotracers, and lack of standardized SUV thresholds. Future large-scale, multicenter, randomized controlled trials are required to validate these findings. Notably, associations between SUV-based parameters and tumor aggressiveness or treatment response are largely correlative rather than causal, and may be influenced by intratumoral heterogeneity, PSMA-low-expressing disease, and treatment-induced modulation of PSMA expression.

### 2.3. Application of PSMA PET/CT in Detecting Lymph Node Metastases of Patients with PCa

Lymph node metastasis (LNM) is primarily identified using CT and MRI, based on morphological features and patterns of enhancement. However, tiny metastatic foci often occur in lymph nodes that are of normal size or only mildly enlarged, resulting in insufficient sensitivity. In addition, inflammatory or reactive lymph node enlargement can cause false-positive findings, further reducing diagnostic specificity. Therefore, CT and MRI are limited in detecting early or microscopic LNMs, which has accelerated the clinical adoption of molecular imaging modalities, such as PSMA PET/CT.

PSMA PET/CT is able to stage PCa LNMs and is characterized by high specificity and a positive predictive value (PPV). A systematic review by Mazzone et al. [53] showed that PSMA PET/CT had a specificity of 94% in identifying LNM. However, its sensitivity was only 54%, indicating it cannot unilaterally guide the intraoperative decision for extended pelvic lymph node dissection (ePLND). Further subgroup analyses in reviews by Petersen et al. and Stabile et al. showed that, while a negative PSMA PET/CT result might reduce unnecessary ePLND in some low-risk patients, a negative scan is still inadequate to safely rule out LNM in high-risk patients [54,55].

Multiple studies have simultaneously investigated the diagnostic performance of different PSMA tracers in detecting LNMs in PCa, as summarized in Table 1. In a multicenter phase III prospective trial, Hope et al. [56] reported that ^68^Ga-PSMA-11 PET/CT achieved a sensitivity of approximately 40% and a specificity as high as 95%, with a PPV of 75% and an NPV of 81% (NCT03368547, NCT02611882, and NCT02919111). In a single-center cohort study, Rajwa et al. [57] found that ^68^Ga-PSMA-11 PET/CT had a sensitivity, specificity, and accuracy of 63%, 97%, and 83%, respectively, in detecting LNM in patients with high-risk nonmetastatic PCa, indicating strong clinical utility in this population. Another small-sample study (*n* = 40) showed that ^68^Ga-PSMA I&T PET/CT could identify LNM with a very high specificity of 98.4%, with an overall accuracy of 93% [37]. In addition, Ingvar et al. [58] observed that although ^18^F-PSMA-1007 PET/CT had an overall sensitivity of only 26.9% for all nodal metastases, the sensitivity increased to 53.8% for lesions with diameter ≥ 3 mm, while maintaining a specificity of 96%. Similarly, the SALT trial by Jansen et al. [59] evaluated the diagnostic accuracy of ^18^F-DCFPyL PET/CT in preoperative lymph node staging (N staging). The results showed a specificity of 94%; however, it may miss micro-metastases due to its limited sensitivity (41.2%). In Cohort A of the OSPREY study [60], ^18^F-DCFPyL PET/CT for LNM detection in preoperative high-risk PCa patients exhibited high specificity (97.9%) and PPV (86.7%), but the sensitivity was only 40.3% (NCT02981368).

In summary, the value of PSMA PET/CT for PCa lymph node staging is mainly reflected by its high specificity and strong confirmatory ability. It can be used to optimize treatment decisions (such as surgical extent or radiation fields). Although it represents an improvement over CT/MRI in detecting micro-lesions, it is still limited by insufficient sensitivity. Therefore, in current clinical practice, PSMA PET/CT is more suitable as a confirmatory tool than as an exclusionary test, and a negative result must be comprehensively assessed by combining it with clinical risk stratification and pathological findings.

### 2.4. The Value of PSMA PET/CT in BCR and mCRPC

Blood biomarkers play a key role in the comprehensive management of PCa, particularly in monitoring and diagnosing BCR and metastatic CRPC (mCRPC). PSA remains the most critical serological indicator. A sustained increase is often the earliest sign of BCR. A sustained increase is often the earliest sign of BCR. The criterion for BCR is defined as a PSA ≥ 0.2 ng/mL on two consecutive measurements following radical prostatectomy, while the standard is PSA Nadir + 2 ng/mL following radiotherapy [61,62,63]. However, PSA alone cannot differentiate between local recurrence and distant metastasis, nor can it reflect the molecular characteristics of the tumor, thus limiting its utility in precise disease classification and prognostic assessment.

PSMA PET/CT can detect recurrent lesions at extremely low PSA levels (even < 0.5 ng/mL), showing a marked advantage over conventional imaging methods and enabling early “restaging” of the disease [64,65]. Guidelines (2024) explicitly state that PSMA-PET/CT is the most sensitive restaging method during the BCR phase and can directly replace routine CT/bone scans, rather than merely serving as a supplement [66]. The CONDOR study which used the “correct localization rate” as its primary endpoint demonstrated that ^18^F-DCFPyL PET/CT was able to identify positive lesions in patients whose conventional imaging (18F-fluciclovine or ^11^C-choline PET, CT, MRI, and/or whole-body bone scintigraphy) results were negative, thereby directly influencing clinical decision-making (NCT03739684) [67]. Although the overall detection rate of PSMA PET/CT did not exceed 50% when PSA < 0.5 ng/mL, it was still significantly higher than that of choline-based PET imaging (12.5%) or conventional CT/bone scans. Furthermore, when PSA levels rose to ≥0.2 ng/mL, the detection rate increased notably to 91.7% [68,69]. Yilmaz et al. [70] and Mena et al. [71] demonstrated a strong correlation between PSA levels and lesion positivity using ^68^Ga-PSMA-I&T and ^18^F-DCFBC, respectively. For ^68^Ga-PSMA-I&T, the positivity rate increased from 43.8% (PSA < 0.2 ng/mL) to 100% (>3.5 ng/mL); for ^18^F-DCFBC, the rates were 15% (PSA < 0.5 ng/mL), 46% (0.5~1.0 ng/mL), 83% (1.0~12.0 ng/mL), and 77% (≥2.0 ng/mL), respectively.

Enhancing the precision and efficacy of salvage radiotherapy (SRT) remains a major clinical challenge in patients with BCR following radical prostatectomy. A phase II randomized clinical trial (NCT03525288) by Belliveau et al. [72] was the first prospective randomized controlled study to validate the clinical value of PSMA PET-guided intensification of salvage radiotherapy (PSMAiSRT). A total of 128 patients with BCR after prostatectomy (median PSA, 0.3 ng/mL) were randomized to receive either standard-of-care SRT (SOC-SRT) or PSMAiSRT. After a median follow-up of 37 months, failure-free survival (FFS) was significantly improved in the PSMAiSRT arm compared with the control arm (hazards ratio (HR) = 0.50; *p* = 0.04), and a similar advantage was observed for eugonadal FFS (HR = 0.45; *p* = 0.03). Subgroup analysis revealed the most pronounced benefit among patients with baseline PSA ≥ 0.3 ng/mL (HR = 0.17; *p* = 0.01). Notably, no significant differences were observed in treatment-related toxicity or quality of life between the groups, confirming that PSMAiSRT enhanced disease control without compromising tolerability. This trial provides robust clinical evidence supporting the incorporation of PSMA-PET imaging in SRT planning for recurrent PCa, underscoring its pivotal role in precision radiotherapy and its potential to optimize patient outcomes.

In patients with mCRPC, PET/CT provides valuable objective and quantitative information regarding tumor burden and serves as an important complement to blood biomarkers for assessing disease progression and treatment response. Kleiburg et al. [73] conducted a retrospective analysis of 60 patients with mCRPC and compared the performance of PSMA PET/CT with that of PSA in evaluating treatment response and predicting survival; 47% of patients showed discordance between PSMA PET/CT and PSA responses. Among them, the vast majority (89%) showed disease progression on PSMA PET/CT, whereas the change in PSA was not significant or even suggested remission. Importantly, the overall response assessed using PSMA PET/CT was an independent predictor of overall survival (OS) (progression vs. non-progression: HR = 4.05; *p* < 0.001), demonstrating superior predictive ability compared to PSA response (progression vs. non-progression: HR = 2.53; *p* = 0.01). Notably, even among patients whose PSA declined by >50%, 31% were still classified as having disease progression based on PSMA PET/CT, and this group demonstrated a significantly higher risk of death (HR = 4.38; *p* = 0.008). This study is the first to systematically demonstrate that PSMA PET/CT can detect true disease progression earlier than PSA during scheduled follow-up evaluations, thus providing a more reliable basis for timely treatment modification. Gafita et al. [74] developed a novel framework known as the Response Evaluation Criteria in PSMA PET/CT (RECIP), which is based on two core criteria: dynamic changes in total tumor volume as measured by PSMA PET/CT and the appearance of new lesions. In a multicenter retrospective study of 124 patients with mCRPC treated with ^177^Lu-PSMA radioligand therapy (RLT), stratification based on RECIP 1.0 showed a strong correlation with PSA progression-free survival (PFS). The median PSA-PFS for the RECIP partial response (PR), stable disease (SD), and disease progression (PD) groups were 8.4, 6.4, and 2.6 months, respectively, which were significantly different (*p* < 0.001).

In 2023, Gafita et al. [75] further validated the inter-reader agreement of the RECIP 1.0 framework (kappa = 0.81) and the consistency between visual assessment and quantitative software analysis (kappa = 0.89). The study also confirmed that RECIP 1.0-defined PD was significantly associated with OS (HR = 2.6; *p* < 0.001), suggesting that RECIP 1.0 is a robust tool for monitoring treatment and predicting prognosis in patients with mCRPC. Shagera et al. [76] extended the application of RECIP 1.0 to patients with mCRPC treated with AR pathway inhibitors (ARPIs) and compared it with conventional PET response assessment criteria. The study found that regardless of the assessment method, patients with a PSMA PET/CT-defined imaging response had significantly longer OS (PSMA responders vs. non-responders: 54 vs. 22 months). Both evaluation methods demonstrated comparable predictive performance (C-index ~0.76–0.79), but RECIP 1.0 offered a more structured and broadly applicable framework.

## 3. Targeting PSMA in PCa Treatment

**Figure 2:**
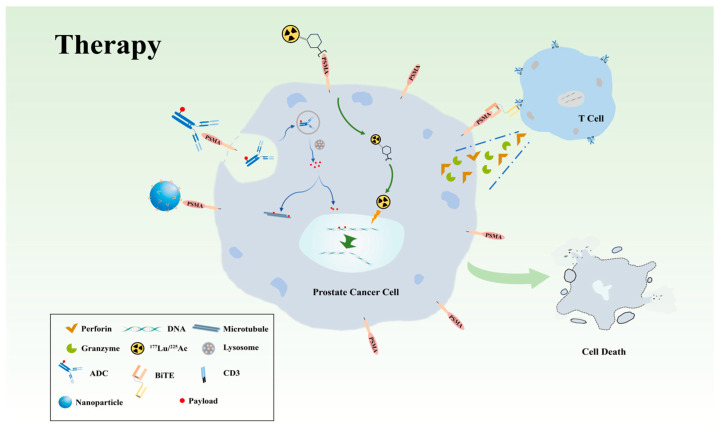
Schematic representation of PSMA-targeted therapeutic strategies and mechanisms in PCa.

The development of PSMA-based fluorescent probes and targeted therapies has paved new avenues for treating PCa at various stages, establishing a paradigm for theranostics (diagnosis and therapy) and precision medicine. As briefly illustrated in Figure 2, targeted therapies such as ^177^Lu-PSMA, PSMA-conjugated nanoparticles, and PSMA antibody–drug conjugates (ADCs) act like “biological missiles,” delivering radionuclides or chemotherapeutic agents directly to PSMA-expressing cancer cells for precise cytotoxicity. Benefiting from the wide expression spectrum of PSMA across different stages of PCa, and even elevated expression levels in advanced/metastatic disease, these approaches offer a valuable option for selected populations or those who have failed multiple lines of treatment, potentially prolonging survival and improving the quality of life of this subset.

### 3.1. PSMA-Targeted Intraoperative Imaging for Precision Resection

Recently, PSMA-based near-infrared (NIR) fluorescence imaging has been validated clinically, demonstrating its unique value for robot-assisted radical prostatectomy (RARP) and pelvic lymph node dissection.

The probe IS-200 couples a PSMA-targeting peptide with an NIR polymethine cyanine dye. Nguyen et al. [77] first confirmed its safety and in vivo clearance profile in a human study. Researchers conducted a phase I trial in high-risk patients undergoing RARP with extended pelvic lymph node dissection. Twenty-four patients were divided into four dose groups: 25, 50, 100, and 150 μg/kg. The results confirmed that at the 25 μg/kg dose, IS-200 clearly visualized PCa foci and residual disease in the surgical bed, achieving an NPV of 100% and a PPV of 80% for detecting residual lesions. This finding suggests its crucial value in intraoperative surgical margin assessment and visualization of residual tumors. Another small-molecule probe, OTL78, comprising a high-affinity PSMA-targeted ligand covalently linked to the NIR dye S0456, was evaluated in a phase IIa clinical study. The optimal regimen was determined to be an injection of 0.03 mg/kg administered 24 h preoperatively. This approach enhances the real-time visualization of primary lesions, resection margins, and surgical bed residues. Histopathological confirmation showed high concordance between intraoperative fluorescence-positive areas and tissue findings [78]. The antibody fragment-based probe IR800-IAB2M derived from J591 was tested for its first human application. It demonstrated a sensitivity and specificity of 100% and 65%, respectively, in detecting peri-prostatic lesions. However, the sensitivity and specificity of lymph node detection were both 64% [79].

Considerable progress has been made in PSMA-targeted dual-modality imaging technologies in recent years. Banerjee et al. [80] and Lütje et al. [81] both applied ^111^In and IRDye800CW for dual-modality imaging, but with different targeting platforms; the former synthesized ^111^In based on a Glu–urea–Lys core as the PSMA-binding motif, while the latter used the PSMA-targeting monoclonal antibody D2B to construct ^111^In-DTPA-D2B-IRDye800CW. In tumor-bearing mice, high-contrast imaging can be achieved as early as 1–2 h after injection of ^111^In. In contrast, because of the higher molecular weight and longer half-life of the antibodies, ^111^In-DTPA-D2B-IRDye800CW imaging can be performed 48 h postinjection. Despite these differences, both probes successfully combined the deep tissue penetration of SPECT for preoperative lesion localization and staging with the high-resolution capability of NIR fluorescence (NIRF) for intraoperative tumor margin delineation, demonstrating the feasibility of PSMA-targeted dual-modality imaging. Furthermore, Schottelius et al. [82] and Eder et al. [83] developed novel PET/NIRF small-molecule probes, PSMA-I&F and PSMA-927, respectively, based on the clinically validated platforms PSMA-I&T and PSMA-617. PSMA-I&F was conjugated to the NIR dye Sulfo-Cy5, whereas PSMA-927 retained IRDye800CW. Experimental studies confirmed that the incorporation of fluorescent dyes did not compromise the high affinity and specificity of PSMA-I&T and PSMA-617 for PSMA, while enabling complementary imaging capabilities.

^68^Ga-P3 is a PSMA-targeted dual-modality probe based on an ODAP-urea scaffold designed to integrate PET and fluorescence imaging capabilities to provide advantages in both preoperative localization and intraoperative navigation. Li et al. [84] synthesized four ODAP-urea dual-modality probes with varying hydrophilicity gradients and systematically compared their affinities, cellular uptake rates, and in vivo imaging performance. All probes demonstrated nanomolar-level PSMA-binding affinity (Ki ≈ 2–4 nM), with the P3 variant (^68^Ga-P3) showing the best in vivo performance. One hour postinjection, clear accumulation was observed in PSMA-positive tumors (SUV_max_ = 1.88 ± 0.36), with a tumor-to-muscle ratio of approximately 12.56 ± 2.63. At 24 h, strong NIRF signals persisted (tumor-to-background: 11.63 ± 4.16), facilitating intraoperative visualization and localization. Building on this, Chen et al. [85] conducted the first in-human clinical study of ^68^Ga-P3 in 2025 to evaluate its safety, imaging efficacy, and feasibility for intraoperative navigation. Multiple cohorts of patients with PCa received intravenous injections of ^68^Ga-P3 at doses of 10, 20, or 40 µg/kg, followed by PET/CT imaging at multiple time points (30, 60, and 120 min), and underwent RARP 24 ± 6 h later. The study reported a PET/CT sensitivity of 79.1%, a specificity of 90.4%, a PPV of 81.5%, and an NPV of 89.0%. Compared with pathological findings, intraoperative fluorescence guidance achieved an overall accuracy of 90.9% and an NPV of 100%, indicating that the probe could effectively guide tumor margin delineation and detect residual lesions intraoperatively. No serious adverse events (SAEs) were reported, demonstrating favorable safety and tolerability.

Moreover, PSMA NIR probes can be combined with traditional white-light- and radiotracer-guided methods. Emerging strategies, including PET/NIR or SPECT/NIR dual-modality probes and their combination with photodynamic or photothermal therapies, are under continuous development. These approaches suggest that PSMA-targeted technology is evolving toward multimodal, integrated diagnosis and therapy, enabling seamless transitions from precise preoperative staging to real-time intraoperative guidance and individualized postoperative treatment [86,87,88]. Nevertheless, further optimization of probe dosing, imaging timing, and imaging equipment is required to improve the detection of minute metastases.

### 3.2. PSMA-Targeted Nuclear Medicine Therapy in Routine Clinic

PSMA acts as a glutamate carboxypeptidase, catalyzing the hydrolysis of extracelluar folate polyglutamates and N-acetylaspartylglutamate [89]. This enzymatic activity increases local glutamate availability and activates oncogenic signaling pathways, including the phosphoinositide 3-kinase (PI3K)/protein kinase B (AKT)/mammalian target of rapamycin (mTOR) signaling axis [90], thereby conferring a selective growth advantage that promotes tumor cell proliferation, metastatic potential, and adaptation to androgen-deprived conditions, ultimately contributing to the development of CRPC [91,92]. These findings establish PSMA as a biologically active and therapeutically actionable target in advanced PCa.

#### 3.2.1. Key Clinical Trials and Evidence of Efficacy of ^177^Lu-PSMA-617

In the field of targeted radionuclide therapy for PCa, clinical studies focusing on PSMA as the core target have advanced rapidly. ^177^Lu-PSMA-617, a representative β-particle therapy, has confirmed its efficacy in multiple high-level randomized controlled trials, as summarized in Table 2.

The phase III VISION study [93] demonstrated that, compared with standard therapy alone, ^177^Lu-PSMA-617 combined with standard care significantly improved both radiographic PFS (rPFS) (median 8.7 vs. 3.4 months; HR = 0.40; *p* < 0.001) and OS (median 15.3 vs. 11.3 months; HR = 0.62; *p* < 0.001). The PSMAfore trial [94] further evaluated the efficacy of ^177^Lu-PSMA-617 in patients with mCRPC who had progressed after one ARPI but had not yet received chemotherapy. The primary endpoint was rPFS. Results showed that compared with patients who switched to another ARPI, ^177^Lu-PSMA-617 significantly prolonged rPFS (median 9.30 vs. 5.55 months; HR = 0.41; *p* < 0.001). An updated analysis showed that this benefit extended to 11.6 months over 5.6 months. The TheraP trial [95] (phase II, NCT03392428) was the first to directly compare ^177^Lu-PSMA-617 with the standard chemotherapy drug Cabazitaxel, and it showed that ^177^Lu-PSMA-617 was superior to Cabazitaxel in terms of PSA50 response rate (66% vs. 37%), radiographic response rate, and maintenance of quality of life. Building on these results, a post hoc analysis of the TheraP trial [96], involving 200 enrolled patients, validated a previously established predictive model for ^177^Lu-PSMA-617 efficacy, aiming to determine whether it could distinguish the patients who would derive greater benefits from ^177^Lu-PSMA-617 compared to chemotherapy. According to findings, the classification model predicting a ≥50% PSA decline not only had prognostic value but also demonstrated predictive utility. Among patients identified by the model as having a favorable prognosis, the PSA50 response rate was significantly higher in the ^177^Lu-PSMA-617 than in the Cabazitaxel group (70% vs. 36%). This result suggests that the model can effectively differentiate patients’ responses to the two treatments and thereby enable clinicians to identify those most likely to achieve a BCR to ^177^Lu-PSMA-617, among patients with mCRPC who progressed after Docetaxel therapy. The PACAP study [97], a real-world multicenter investigation, enrolled patients with mCRPC who had previously received Cabazitaxel to evaluate the efficacy and prognostic factors of ^177^Lu-PSMA-617 therapy. The results showed that after treatment with ^177^Lu-PSMA-617, 44% of patients achieved a ≥50% PSA decline, and the objective response rate (ORR) was 35%. However, the median rPFS was only 4.4 months, indicating that the duration of response was limited. The analysis also identified several key prognostic factors: patients whose time to the development of castration resistance was less than 12 months had a shorter median rPFS than their counterparts (3.1 vs. 4.8 months; HR = 1.77, 95% CI [1.14–2.77]). In contrast, a PSA decline ≥50% was significantly associated with longer rPFS, time to PSA progression (TTPSA), and OS. For example, patients who achieved a PSA reduction ≥50% had a median rPFS of approximately 9.0 months, compared with only 2.7 months in those without a PSA decline (*p* < 0.001).

The UpFrontPSMA phase II clinical trial (NCT04343885) [98] focused on patients with newly diagnosed high-tumor-burden metastatic hormone-sensitive prostate cancer (mHSPC), comparing sequential administration of ^177^Lu-PSMA-617 followed by Docetaxel versus Docetaxel alone. The primary endpoint was the proportion of patients achieving a PSA level ≤ 0.2 ng/mL at 48 weeks after treatment initiation. Results showed that at 48 weeks, a significantly higher proportion of patients in the experimental arm achieved the target PSA level compared with the control group (41% vs. 16%, *p* = 0.002).

In summary, collective evidence obtained from the VISION trial, which established ^177^Lu-PSMA-617 as a standard of care, the TheraP trial, which highlighted its comparative advantages and post hoc precision stratification, the PSMAfore trial, demonstrating earlier-line application, the PACAP real-world validation, and finally, the UpFrontPSMA exploration in hormone-sensitive disease underscores the expanding clinical role and future potential of ^177^Lu-PSMA-617 RLT in the comprehensive management of PCa (Table 2).

**Table 2:** **Clinical study of ^177^Lu-PSMA-617 in patients with mCRPC and mHSPC.**

Study	Phase	Study Population	Study Protocol	Primary End Point	Main Results	NCT#
VISION [93]	III	pre-treated mCRPC	^177^Lu-PSMA-617 + standard-care therapy vs. standard-care therapy	rPFS, OS	rPFS: 8.7 vs. 3.4 months	NCT03511664
					OS: 15.3 vs. 11.3 months	
PSMAfore [94]	III	mCRPC, ARPI failure and taxane-naive	^177^Lu-PSMA-617 vs. ARPI change	rPFS	9.3 vs. 5.6 months	NCT04689828
TheraP [95]	II	pre-treated mCRPC and PSMA-positive	^177^Lu-PSMA-617 vs. Cabazitaxel	PSA50 response	66% vs. 37%	NCT03392428
PACAP [97]	-	mCRPC pre-treated with cabazitaxel	^177^Lu-PSMA-617	rPFS	4.4 months	-
UpFrontPSMA [98]	II	mHSPC	sequential ^177^Lu-PSMA-617 and Docetaxel vs. Docetaxel	PSA ≤ 0.2 ng/mL at 48 weeks	41% vs. 16%	NCT04343885

mCRPC: metastatic castration-resistant prostate cancer; mHSPC: metastatic hormone-sensitive prostate cancer; rPFS: radiographic progression-free survival; OS: overall survival; PSA: prostate-specific antigen; ARPI: androgen receptor pathway inhibitor; PSA 50: PSA reduction of at least 50% from baseline.

#### 3.2.2. Emerging Role of ^225^Ac-PSMA-617 in α-Particle RLT

α particles possess high energy and a short tissue penetration range, allowing them to effectively kill cancer cells while minimizing damage to surrounding healthy tissues. In the field of α-particle RLT, ^225^Ac-PSMA-617 has demonstrated notable antitumor activity and unique clinical potential. We summarize those trials’ endpoints as well as adverse events in Table 3.

A pilot study in patients with advanced metastatic PCa who had not received prior chemotherapy demonstrated that ^225^Ac-PSMA-617 achieved a high PSA response rate; PSA levels were reduced by >90% in 82% of patients, and serum PSA was undetectable in some patients. Furthermore, a >50% reduction in lesion avidity was observed in 15 patients using ^68^Ga-PSMA PET/CT imaging [99]. In patients with mCRPC, Sathekge et al. [100] provided pivotal retrospective evidence that ^225^Ac-PSMA-617 remained effective even after multiple prior therapies, including ADT. In their cohort, 96% of patients experienced PSA decline, and 91% achieved PSA reductions > 50%. The multicenter WARMTH Act study [101] encompassing seven international centers further validated these findings in a large sample of patients with mCRPC who had received prior treatments with Docetaxel, Cabazitaxel, or ^177^Lu-PSMA-617. Patients who received one or more cycles of identical doses of ^225^Ac-PSMA-617 achieved a median OS of approximately 15.5 months, with over half of them reaching PSA50 response, confirming the broad efficacy of ^225^Ac-PSMA-617 across diverse treatment histories. In addition, Feuerecker et al. [102] and Yadav et al. [103] found that in patients who had failed or developed resistance to ^177^Lu-PSMA-617 therapy, ^225^Ac-PSMA-617 continued to demonstrate strong efficacy and acceptable safety. In the former study, the median PSA-PFS and OS were 3.5 months and 7.7 months, respectively. In the latter study, the median PFS and OS were 12 months and 17 months, respectively. Both studies highlighted the potential of ^225^Ac-PSMA-617 as a salvage therapy for advanced PCa. However, these studies also highlighted the challenge of salivary gland toxicity, a frequent adverse effect that limits its broader use. To mitigate this, a single-center study involving 233 patients compared dose de-escalation and ^177^Lu/^225^Ac combination (“cocktail”) therapies, finding that both approaches maintained high PSA response rates while significantly reducing xerostomia, suggesting that dose optimization and dual-isotope strategies may improve tolerability [104]. Khreish et al. [105] used a similar strategy of combining ^177^Lu and ^225^Ac but with a different sequence; they administered a low dose of ^225^Ac-PSMA-617 first, followed by a standard dose of ^177^Lu-PSMA-617 over two consecutive days within the same week. This regimen achieved comparable therapeutic efficacy while reducing salivary gland toxicity, offering a promising new approach that leverages the complementary radiobiological advantages of α and β particles.

#### 3.2.3. Safety Concerns and Eligible Population

PSMA-targeted radioligand therapies exhibit distinct but largely predictable safety profiles that influence treatment feasibility. For β-emitting agents, particularly ^177^Lu-PSMA-617, cumulative bone marrow toxicity represents the primary factor limiting treatment continuity. Large cohort studies demonstrate that clinically significant hematologic adverse events are relatively infrequent and mostly reversible but occur more commonly in patients with extensive bone metastases, pre-existing cytopenias, or prior taxane chemotherapy, which may restrict the completion of multiple treatment cycles [106]. Nevertheless, ^177^Lu-PSMA therapy remains clinically feasible across both heavily pre-treated and taxane-naïve populations, with earlier use associated with improved survival outcomes and preserved tolerability [107]. Renal toxicity is uncommon and generally mild, with long-term studies and dosimetry data confirming stable renal function and cumulative absorbed doses below established safety thresholds during repeated cycles [108,109].

In contrast, α-emitting ^225^Ac-PSMA therapy, while highly potent, is predominantly constrained by salivary gland toxicity. Xerostomia is reported in the majority of treated patients and frequently necessitates dose de-escalation or treatment modification to maintain quality of life, thereby defining the principal dose-limiting toxicity of α-based PSMA therapies.

Collectively, β-therapy is primarily constrained by cumulative marrow toxicity, whereas α-therapy is limited by salivary gland injury, together with additional challenges such as PSMA-low or heterogeneous tumors and constraints on long-term or repeated treatment, underscoring the need for optimized dosing paradigms, careful patient selection, and tailored toxicity mitigation strategies to balance efficacy and tolerability.

### 3.3. Novel PSMA-Targeted Therapy Under Investigation in PCa

PSMA-targeted radioligand therapies, particularly ^177^Lu-PSMA and ^225^Ac-PSMA, have established a solid clinical foundation for precision treatment of PCa, while also highlighting key limitations related to toxicity, therapeutic window, and tumor heterogeneity. In response, the field is expanding toward next-generation PSMA-directed strategies that incorporate alternative delivery systems and immune-based mechanisms to enhance tumor specificity and versatility.

#### 3.3.1. Multifunctional PSMA-Targeted Nanoplatforms: From Drug Delivery to Integrated Therapy

In recent years, researchers have developed several strategies to drive the diversification of PSMA-targeted nanoplatforms to meet different needs such as drug delivery, imaging guidance, photodynamic/photothermal therapy, combination therapy, and immunomodulation. By conjugating small-molecule inhibitors, aptamers, or antibody fragments to polymeric nanocarriers, these systems enable precise delivery of chemotherapeutic agents or peptide molecules, offering new possibilities for integrated diagnosis and therapy.

Owing to their high specific surface area and ease of surface modification, Mesoporous Silica Nanoparticles (MSNs) are utilized to deliver classic chemotherapeutic drugs, such as Paclitaxel. Rivero-Buceta et al. [110] designed a PSMA-targeted MSN to encapsulate the chemotherapeutic drug Docetaxel and validated its efficacy in various PCa cell lines. The study showed that this nanoparticle exhibited high targeting specificity—its intracellular uptake in LNCaP cells increased by 25% compared to non-targeted delivery systems (*p* < 0.001). Additionally, its cytotoxic activity was enhanced by two orders of magnitude relative to that of the free drug.

Metallic nanoparticles have been widely explored owing to their excellent optical and photothermal properties as well as modifiable surfaces. Mangadlao et al. [111] reported a PSMA-targeted gold nanoparticle that utilized a PSMA ligand conjugated to the gold nanoparticle surface to achieve specific recognition and targeted delivery to PCa cells. This system functions not only as a fluorescent imaging probe for precise tumor visualization but also as an agent for photodynamic therapy (PDT) under external laser irradiation, selectively killing PSMA-positive cells. In vivo experiments have demonstrated effective tumor accumulation in a mouse PCa model and significant tumor suppression after light exposure, highlighting its advantages over traditional chemotherapy in theranostic integration. Similarly, PSMA-targeted melanin-like nanoparticles incorporating the photosensitizer Ce6 and the imaging agent perfluoropentane (PFP) have been shown to synergistically combine photothermal therapy (PTT) and PDT for enhanced tumor ablation, while amplifying ultrasound imaging signals for real-time tumor monitoring. These nanoparticles exhibited excellent biocompatibility and low toxicity [112]. Chen et al. [113] proposed an innovative NIR-II image-guided self-enhanced nanomedicine capable of generating oxygen in an acidic tumor microenvironment and producing multiple reactive oxygen species (ROS). This platform leveraged the high tissue penetration and precision of NIR-II fluorescence imaging to overcome the hypoxia limitations of conventional photodynamic therapy, thereby significantly improving therapeutic efficacy in PCa.

All the aforementioned studies involved single-drug delivery, whereas Adekiya et al. [114] developed a PSMA-targeted nanoparticle capable of simultaneously delivering Docetaxel and brusatol to achieve synergistic therapy. Guided by a surface-conjugated PSMA-targeting ligand, these nanoparticles exhibited enhanced selective uptake by PSMA-positive LNCaP cells and significantly increased intracellular ROS levels, thereby promoting cancer cell apoptosis. In vitro studies demonstrated that these nanoparticles showed markedly superior cytotoxicity against LNCaP cells compared with single-drug or non-targeted nanoparticles. In vivo mouse model experiments further confirmed the high tumor accumulation and enhanced antitumor efficacy, accompanied by reduced systemic toxicity. Another study utilized polyethylene glycol-polylactic acid (PEG-PLA) nanoparticles for targeted co-delivery of galbanic acid and Docetaxel, demonstrating significantly improved cellular uptake efficiency and enhanced drug release in an acidic microenvironment (pH 5.5) [115]. To address the drug resistance and immunosuppression commonly observed in advanced PCa, Yin et al. [116] designed glutathione-responsive PSMA-targeted nanoparticles (PSMA NP/BEZ) encapsulating the PI3K/mTOR dual inhibitor prodrug BEZ235. By specifically inhibiting the PI3K/mTOR pathway, these nanoparticles not only overcome multidrug resistance (via downregulation of P-glycoprotein) but also alleviate immune suppression (through inactivation of myeloid-derived suppressor cells, MDSCs), thereby markedly improving both chemotherapeutic and immunotherapeutic efficacy in advanced PCa. 

PSMA-targeted nanoparticles have been clinically evaluated: BIND-014 was the first PSMA-targeted nanomedicine to enter clinical trials with Docetaxel as its core payload. In a phase I trial (NCT01300533) [117] involving 31 patients with advanced solid tumors (including prostate, lung, and gastric cancers), the MTD was determined to be 60 mg/m^2^. The main toxic effects were similar to those of free Docetaxel, although the myelosuppression was mild. Some patients achieved disease stabilization, and one patient with PCa showed a partial response. The phase II trial (NCT01812746) [118], conducted in chemotherapy-naïve patients with mCRPC, enrolled 42 participants, among whom 30% achieved PSA reductions ≥50%. Moreover, PSMA-positive CTCs were preferentially eliminated following treatment. The toxicity profiles were consistent with phase I results. Collectively, these clinical trials demonstrate that BIND-014 can provide disease control and clinical benefits in patients with PCa.

#### 3.3.2. Towards Precision Medicine: PSMA-Targeted ADCs

PSMA has become a crucial target in the development of ADCs for PCa owing to its high tumor-specific expression in PCa cells. By conjugating an anti-PSMA monoclonal antibody with a cytotoxic small-molecule drug, PSMA-ADCs can selectively deliver the toxin to cancer cells, thereby enhancing the therapeutic efficacy and reducing systemic toxicity.

Early studies, exemplified by the PSMA-targeted ADC, MLN2704, fully validated the potential of this therapeutic strategy [119]. MLN2704 was constructed by conjugating the deimmunized J591 antibody (MLN591) with the drug maytansinoid 1 (DM1) to achieve “precise drug delivery”. This conjugate selectively binds to PSMA, inducing its internalization into cancer cells where it subsequently releases DM1 to exert its cytotoxic effects. The preclinical data are encouraging. MLN2704 exhibited favorable pharmacokinetic properties and excellent tumor penetration. An optimized dosing regimen resulted in prolonged tumor growth delay of up to approximately 100 days in mouse xenograft models. This study also emphasized the importance of target specificity, as the unconjugated antibody or free drug alone showed minimal activity. Furthermore, MLN2704 demonstrated efficacy in a mouse model of bone metastasis of PCa. These findings establish the therapeutic validity of the PSMA-targeted ADC approach and lay a solid foundation for the development of new-generation, highly selective drugs, particularly for treating mCRPC. A phase I clinical trial [120] of MLN2704 in 37 patients with progressive mCRPC showed that some patients achieved a PSA decline ≥ 50% and disease stabilization, indicating clear biological activity. The primary dose-limiting toxicity (DLT) was dose-dependent neuropathy. A subsequent phase I/II dose-escalation study [121] in 62 patients with mCRPC confirmed that neurotoxicity remained the principal dose-limiting factor; 71% of patients experienced peripheral neuropathy, including 10% with Grade 3/4. Pharmacokinetic analyses revealed instability of the disulfide linker, leading to rapid deconjugation of DM1, which is believed to cause neurotoxicity. As only 8% of patients achieved a sustained PSA decline ≥ 50% and the therapeutic window was narrow, the agent showed limited clinical activity in mCRPC, and further development of the agent was discontinued.

PSMA-ADC is structurally based on a fully human IgG1 monoclonal antibody targeting PSMA, conjugated via a cleavable linker to the microtubule-disrupting agent monomethyl auristatin E (MMAE). In a phase I dose-escalation trial (NCT01414283) [122], a total of 52 patients with progressive mCRPC were enrolled to evaluate the safety, pharmacokinetics, and preliminary efficacy of PSMA-ADC. The MTD was determined to be 2.5 mg/kg administered once every 3 weeks, with DLTs including neutropenia and reversible liver function abnormalities. Peripheral neuropathy is a notable late-onset toxicity. Among patients receiving doses ≥1.8 mg/kg, eight patients had PSA declines of ≥50%, and in those receiving ≥1.6 mg/kg, eight patients showed a reduction in CTC counts, indicating preliminary antitumor activity. These findings suggest that PSMA-ADC has acceptable toxicity and measurable antitumor efficacy in heavily pre-treated (chemotherapy regimens and abiraterone and/or enzalutamide) patients with mCRPC, providing essential safety and dosing data for further clinical development. Subsequently, a phase II open-label, single-arm study (NCT01695044) [123] enrolled 119 patients with mCRPC who progressed after abiraterone or enzalutamide therapy. The results showed a PSA50 response rate of approximately 14–21%, with 78% of patients exhibiting a ≥50% reduction in CTC counts. The therapeutic efficacy was found to correlate with high PSMA expression in tumors. The most common clinically significant treatment-related adverse events (TRAEs) include neutropenia, fatigue, electrolyte imbalance, anemia, and neuropathy. SAEs included dehydration, hyponatremia, and febrile neutropenia.

MEDI3726 (ADCT-401) consists of a site-specific conjugate of the human PSMA-targeting antibody J591 and the pyrrolobenzodiazepine (PBD) dimer tesirine. In preclinical models, MEDI3726 demonstrates high selectivity and potent antitumor activity [124]. In vitro, MEDI3726 showed sub-nanomolar IC_50_ values against PSMA-high-expressing cell lines such as LNCaP and 22Rv1, while exhibiting almost no activity against PSMA-negative cells, confirming its high specificity. In in vivo xenograft models, the drug exhibited sustained dose-dependent tumor inhibition in mice. However, in a subsequent phase I clinical trial [125], MEDI3726 achieved only a 12.1% composite response rate, and this effect was observed only at higher dose levels. Moreover, the majority of patients experienced TRAEs, with nearly half of them being Grade 3/4 and 33% of patients discontinuing treatment due to toxicity. Consequently, although preliminary antitumor activity was observed, the study failed to establish an MTD, and the clinical responses were not durable because the patients could not tolerate prolonged dosing. These findings indicate that drug safety and tolerability represent major challenges for the further development of MEDI3726.

ARX517 is an ADC that was developed for treating mCRPC. ARX517 uses AS269 as its payload and achieves site-specific conjugation through an oxime bond to two synthetic amino acids introduced at defined positions on the antibody (drug-to-antibody ratio of 2), thereby minimizing off-target drug release. Preclinical studies [126] demonstrated that ARX517 binds selectively to PSMA and is internalized by cancer cells, where it releases its cytotoxic payload to induce apoptosis. In multiple murine models of mCRPC, both enzalutamide-sensitive and enzalutamide-resistant ARX517 exhibited significant dose-dependent antitumor activity. In non-human primate studies, ARX517 displayed a half-life of 13.5 days, with no evidence of severe neurotoxicity or notable myelosuppression. Only mild and reversible elevations in liver function markers were observed. These findings highlight ARX517’s structural stability, potent selectivity, and favorable safety profile, addressing the toxicity and instability issues observed in earlier PSMA-ADCs and providing a strong rationale for its clinical development.

Based on these findings, the APEX-01 early-phase clinical trial was initiated (NCT04662580) [127]. Preliminary results presented at the 2023 European Society for Medical Oncology (ESMO) Congress showed that no DLTs or treatment-related SAEs were observed across all tested dose levels. The main adverse events were mild to moderate fatigue, dry mouth, diarrhea, and thrombocytopenia, with no severe neurotoxicity typically observed with traditional ADCs. This safety profile is attributed to the stable site-specific conjugation of the agent, which leads to very low off-target payload release. In cohorts receiving a dose of ≥2.0 mg/kg, patients achieved a ≥50% decline in PSA as well as a ≥50% reduction in ctDNA. Overall, ARX517 is the first PSMA-targeted ADC to demonstrate a strong balance between potent antitumor activity and favorable safety in a clinical setting and is regarded as a major breakthrough in this field.

#### 3.3.3. Cancer and Immune Interaction: PSMA-Targeted T-Cell Therapy

Unlike conventional treatment modalities, cancer immunotherapy aims to harness the body’s own immune system to attack tumor cells while establishing long-lasting immune memory. Immunotherapy for PCa has gradually expanded to include PSMA-targeted T-cell therapies, the core concept of which is to exploit the potent cytotoxic capability of T cells to precisely eliminate PSMA-expressing cancer cells.

(1) Bispecific T-cell engagers

Bispecific T-cell engagers (BiTEs) are a class of engineered bispecific antibodies capable of simultaneously binding to a tumor-associated antigen (such as PSMA in PCa) and a CD3 complex on T cells. This bridging mechanism enables the direct recruitment of a patient’s own T cells to the tumor cells. It does not require antigen presentation or involvement of the major histocompatibility complex. Consequently, it triggers T-cell activation, proliferation, and cytotoxic effects that lead to tumor cell apoptosis.

A phase I dose-escalation study [128] of Pasotuxizumab (AMG 212 or BAY 2010112) (NCT01723475) provided the first clinical evidence that BiTE monotherapy can be effective in patients with mCRPC. The drug exhibited clear dose-dependent activity, with the highest dose group achieving an average PSA decline of 54.9%. The main toxicity was cytokine release syndrome (CRS), associated with T-cell activation, which was generally manageable and reversible. Importantly, this study revealed that the route of administration was critical for maintaining efficacy; subcutaneous injection led to a high incidence of antidrug antibody (ADA) formation, whereas continuous intravenous infusion did not, establishing a key dosing paradigm for subsequent PSMA-targeted BiTEs such as Acapatamab.

Acapatamab (AMG 160) is an Fc-engineered, half-life-extended PSMA × CD3 bispecific antibody. In a multicenter phase I trial (NCT03792841) [129], Acapatamab achieved a PSA50 response rate of 30.4% and demonstrated partial radiographic response in some patients. The most common adverse event was CRS (approximately 97%), but it could be effectively managed through dexamethasone premedication, intravenous hydration, and step-up dosing. The extended half-life of Acapatamab significantly improved dosing convenience and treatment feasibility. JNJ-63898081 (JNJ-081) performed slightly worse than the two abovementioned agents in the clinical trial (NCT03926013) [130]. The study enrolled 39 patients with mCRPC. Transient PSA decline occurred when the subcutaneous dose exceeded 30 μg/kg, and only two patients had a PSA decline > 50%. More importantly, up to half of the patients (particularly those in the subcutaneous injection group) developed ADAs, which negatively affected treatment efficacy. Although JNJ-63898081 is feasible for mCRPC treatment, its high immunogenicity limits its clinical activity and durability.

(2) Chimeric antigen receptor T-Cell immunotherapy

High PSMA expression on the surface of tumor cells provides an ideal foundation for chimeric antigen receptor T-cell (CAR-T) therapy. With the maturation of immune cell engineering technologies, PSMA-targeted CAR-T cell therapy has gradually advanced from early proof-of-concept studies to clinical exploration.

In 2016, Junghans et al. [131] published the first clinical study evaluating the safety of PSMA-directed CAR-T cells in humans. This study enrolled patients with multilineage treatment-refractory mCRPC. Without chemotherapy for lymphodepletion, this study relied on exogenous interleukin-2 (IL-2) to enhance CAR-T cell expansion. The results showed that the treatment was generally safe, and two patients achieved clinically meaningful partial responses. No Grade ≥ 3 CRS or neurotoxicity was observed. Some patients exhibited decreased PSA levels and signs of immune activation. This study is the first to emphasize that the pharmacokinetics of the interaction between CAR-T cells and IL-2 is a critical factor for successful solid tumor treatment using cell therapy. At the preclinical stage, Alzubi et al. [132] further demonstrated the potential of combination strategies. Systemic administration of PSMA CAR-T cells or low-dose non-ablative Docetaxel alone was sufficient to inhibit tumor growth; however, their combination significantly suppressed or even eradicated tumors, indicating a strong synergistic effect. Moreover, focal injection of PSMA-targeted CAR-T cells eradicated the established human PCa xenografts, suggesting the potential of CAR-T cells as a localized therapeutic approach. To overcome the immunosuppressive tumor microenvironment, Narayan et al. engineered PSMA-targeted CAR-T cells to co-express a dominant-negative transforming growth factor beta (TGFβ) receptor (TGFβRDN), enabling them to resist TGFβ-mediated immunosuppression. In a phase I clinical trial (NCT03089203) [133] involving patients with mCRPC, this therapy resulted in T cell expansion in the blood and migration to the tumor site. Preliminary efficacy data showed that 40% of patients achieved a PSA decline ≥ 30%. However, CRS remained the primary concern, with one case of Grade 4 severe adverse events.

However, emerging approaches such as nanotheranostic platforms, PSMA-ADCs, PSMA-targeted CAR-T cells, and bispecific T-cell engagers are currently at preclinical or early-phase clinical stages and require further validation to establish their safety, efficacy, and clinical feasibility.

## 4. Applications of PSMA in Non-Prostate Cancer

In recent years, with the successful application of PSMA-targeted imaging and radionuclide therapy in PCa, its potential in non-prostate malignancies has drawn increasing attention. Although PSMA expression is generally lower and more heterogeneous in non-prostate malignancies than in PCa, it is frequently detected in neovascular endothelial cells of various solid tumors. This provides a theoretical foundation for utilizing PSMA as a “vascular target” in combined imaging and therapeutic strategies (e.g., PSMA expression in tumor-associated vasculature) [134]. From a molecular imaging perspective, the application of PSMA-labeled PET/CT has been explored in multiple types of solid tumors. For instance, in salivary gland adenoid cystic carcinoma (ACC), PSMA expression levels are generally high, and PET/CT can clearly visualize primary and metastatic lesions, and in some patients, it is superior to ^18^F-FDG PET/CT, suggesting its role as a supplementary tool for staging and therapeutic assessment [135]. Moreover, varying degrees of PSMA uptake have been observed in renal cell carcinoma, breast cancer, gastrointestinal malignancies, and brain metastases of non-prostatic origin, although the overall signal intensity and detection rates show significant heterogeneity [136,137]. However, the diagnostic accuracy of PSMA PET/CT in non-prostate tumors is further compromised because non-specific PSMA uptake is often associated with neovascularization or tissue repair, leading to false positives in some benign lesions (such as inflammation, granulation tissue, and fracture repair) [138]. In terms of therapy, PSMA-targeted RLT for non-PCAs remains in the exploratory stage. Case reports and small cohort studies have demonstrated that ^177^Lu-PSMA-617 treatment can induce partial metabolic responses or disease stabilization in patients with adenoid cystic carcinoma of the salivary glands, indicating its potential as a novel therapeutic option for this rare malignancy [136]. However, its overall efficacy is limited by insufficient PSMA expression, high intratumoral heterogeneity of uptake, challenges in dosimetry optimization, and constraints in normal organ tolerance.

In summary, the application of PSMA-targeted imaging and therapy in non-PCa holds considerable promise; however, its real value in clinical decision-making and improving prognosis still relies on validation through multicenter prospective studies.

## 5. Conclusions

The landscape of PCa management is being increasingly reshaped by PSMA-targeted strategies. Recent advances in ligand engineering, particularly the development of PSMA-targeted nanobodies, have introduced promising alternatives to conventional monoclonal antibodies such as J591, offering improved pharmacokinetic profiles characterized by faster blood clearance and enhanced tumor penetration. Therapeutic advancement is further exemplified by the clinical transition toward α-particle radiopharmaceutical therapy, which delivers high linear energy transfer and has shown potential to address treatment resistance in mCRPC. In parallel, combinatorial approaches are emerging as a key frontier; pharmacological modulation of the AR signaling axis using next-generation antiandrogens has been shown to upregulate PSMA expression, thereby potentially sensitizing tumors to subsequent PSMA-targeted therapeutic delivery. Looking ahead, PSMA-based strategies are anticipated to move upstream along the clinical continuum, evolving from salvage therapies for advanced disease toward tools for early staging, treatment stratification, and neoadjuvant personalized intervention, ultimately strengthening the translational link between molecular targeting and durable clinical benefit.

## Figures and Tables

**Table 1:** Diagnostic performance of ^18^F and ^68^Ga-labeled PSMA PET/CT for detecting lymph node metastases in PCa.

Author	Study Design	Tracer	NPV	PPV	Sensitivity	Specificity
Cytawa et al. [37]	retrospective	^68^Ga	95.0%	63.60%	35.0%	98.4%
Hope et al. [56]	prospective	^68^Ga	81.0%	75.0%	40.0%	95.0%
			95% CI [76.0–85.0]	95% CI [70.0–80.0]	95% CI [34.0–36.0]	95% CI [92.0–97.0]
Rajwa et al. [57]	retrospective	^68^Ga	79.0%	94.00%	63.00%	97.00%
			95% CI [70.0–86.0]	95% CI [82.0–99.0]	95% CI [51.0–75.0]	95% CI [91.0–99.0]
Ingvar et al. [58]	retrospective	^18^F	79.8%	70.0%	26.9%	96.2%
					95% CI [11.6–47.8]	95% CI [98.2–99.2]
Jansen et al. [59]	prospective	^18^F	90.4%	53.8%	41.2%	94.0%
			95% CI [82.6–95.0]	95% CI [26.1–79.6]	95% CI [19.4–66.5]	95% CI [86.9–97.5]
Pienta et al. [60]	prospective	^18^F	83.2%	86.7%	40.3%	97.9%
			95% CI [78.2–88.1]	95% CI [69.7–95.3]	95% CI [28.1–52.5]	95% CI [94.5–99.4]

NPV: negative predictive value; PPV: positive predictive value; 95% CI: 95% confidence interval.

**Table 3:** **Clinical study on the therapeutic effect of ^225^Ac-PSMA conducted in patients with PCa.**

Study	Study Design	Study Population	Study Protocol	Main Results	Toxicity
Sathekge et al., 2019 [99]	retrospective	chemotherapy-naïve patients with advanced metastatic PCa	dosage de-escalation of ^225^Ac-PSMA-617	14 patients with a PSA decline ≥ 90%;	reduced salivary toxicity
				7 patients with undetectable PSA;	
Sathekge et al., 2022 [100]	retrospective	post-ADT mCRPC patients	^225^Ac-PSMA-617	48/53 patients with a PSA decline > 50%	xerostomia
					nephrotoxicity
Sathekge et al., 2024 [101]	retrospective	pre-treated mCRPC patients	^225^Ac-PSMA-617	median OS: 15.5 months	xerostomia
				median PFS: 7.9 months	bone marrow and renal toxicity
Feuerecker et al., 2025 [102]	retrospective	mCRPC patients post ^177^Lu-PSMA failure	^225^Ac-PSMA-617	median OS: 7.7 months	xerostomia
				median PSA-PFS: 3.5 months	
Yadav et al., 2020 [103]	prospective	mCRPC patients refractory to or naïve for ^177^Lu-PSMA-617	^225^Ac-PSMA-617	median OS: 17 months	xerostomia
				median PFS: 12 months	
Rathke et al., 2023 [104]	retrospective	PCa	^225^Ac-PSMA-617 de-escalated monotherapy vs. cocktail regimen *	PSA decline ≥ 50%: 55 vs. 74 patients	reduced xerostomia
				median OS: 9 vs. 15 months	
Khreish et al., 2020 [105]	retrospective	mCRPC patients with suboptimal response to ^177^Lu-PSMA-617monotherapy	low-activity ^225^Ac-PSMA-617/ full-activity ^177^Lu-PSMA-617 tandem therapy	median OS: 48 weeks	reduced xerostomia
				median PFS: 19 weeks	

mCRPC: metastatic castration-resistant prostate cancer; PFS: progression-free survival; OS: overall survival; PSA: prostate-specific antigen; ADT: androgen deprivation therapy. * ^177^Lu-PSMA-617 plus ^225^Ac-PSMA-617.

## Data Availability

No new data were created or analyzed in this study.

## References

[1] Siegel R.L., Kratzer T.B., Wagle N.S., Sung H., Jemal A. (2026). Cancer Statistics, 2026. CA Cancer J. Clin..

[2] Raychaudhuri R., Lin D.W., Montgomery R.B. (2025). Prostate Cancer: A Review. JAMA.

[3] James N.D., Tannock I., N’Dow J., Feng F., Gillessen S., Ali S.A., Trujillo B., Al-Lazikani B., Attard G., Bray F. (2024). The Lancet Commission on Prostate Cancer: Planning for the Surge in Cases. Lancet.

[4] Khan S., Zakariah M., Palaniappan S. (2016). Computational Prediction of *Mycoplasma hominis* Proteins Targeting in Nucleus of Host Cell and Their Implication in Prostate Cancer Etiology. Tumor Biol..

[5] Khan S., Zakariah M., Rolfo C., Robrecht L., Palaniappan S. (2017). Prediction of *Mycoplasma hominis* Proteins Targeting in Mitochondria and Cytoplasm of Host Cells and Their Implication in Prostate Cancer Etiology. Oncotarget.

[6] Wang Y., Imran A., Shami A., Chaudhary A.A., Khan S. (2021). Decipher the *Helicobacter pylori* Protein Targeting in the Nucleus of Host Cell and Their Implications in Gallbladder Cancer: An Insilico Approach. J. Cancer.

[7] Nyberg T., Frost D., Barrowdale D., Evans D.G., Bancroft E., Adlard J., Ahmed M., Barwell J., Brady A.F., Brewer C. (2020). Prostate Cancer Risks for Male BRCA1 and BRCA2 Mutation Carriers: A Prospective Cohort Study. Eur. Urol..

[8] Ahamed Y., Hossain M., Baral S., Al-Raiyan A.U., Ashraf S.B., Sun W. (2025). The Research Progress on Diagnostic Indicators Related to Prostate-Specific Antigen Gray-Zone Prostate Cancer. BMC Cancer.

[9] Kania E., Janica M., Nesterowicz M., Modzelewski W., Cybulski M., Janica J. (2025). Advances and Challenges in Prostate Cancer Diagnosis: A Comprehensive Review. Cancers.

[10] Shi M.-J., Wang Z.-X., Wang S.-K., Li X.-H., Zhang Y.-L., Yan Y., An R., Dong L.-N., Qiu L., Tian T. (2025). Performance of GPT-4 for Automated Prostate Biopsy Decision-Making Based on mpMRI: A Multi-Center Evidence Study. Mil. Med. Res..

[11] Parsi M., Desai M.H., Desai D., Singhal S., Khandwala P.M., Potdar R.R. (2021). PSMA: A Game Changer in the Diagnosis and Treatment of Advanced Prostate Cancer. Med. Oncol..

[12] Backhaus P., Noto B., Avramovic N., Grubert L.S., Huss S., Bögemann M., Stegger L., Weckesser M., Schäfers M., Rahbar K. (2018). Targeting PSMA by Radioligands in Non-Prostate Disease—Current Status and Future Perspectives. Eur. J. Nucl. Med. Mol. Imaging.

[13] Patell K., Kurian M., Garcia J.A., Mendiratta P., Barata P.C., Jia A.Y., Spratt D.E., Brown J.R. (2023). Lutetium-177 PSMA for the Treatment of Metastatic Castrate Resistant Prostate Cancer: A Systematic Review. Expert Rev. Anticancer Ther..

[14] Naik M., Khan S.R., Lewington V., Challapalli A., Eccles A., Barwick T.D. (2024). Imaging and Therapy in Prostate Cancer Using Prostate Specific Membrane Antigen Radioligands. Br. J. Radiol..

[15] Hupe M.C., Philippi C., Roth D., Kümpers C., Ribbat-Idel J., Becker F., Joerg V., Duensing S., Lubczyk V.H., Kirfel J. (2018). Expression of Prostate-Specific Membrane Antigen (PSMA) on Biopsies Is an Independent Risk Stratifier of Prostate Cancer Patients at Time of Initial Diagnosis. Front. Oncol..

[16] Sayar E., Patel R.A., Coleman I.M., Roudier M.P., Zhang A., Mustafi P., Low J.-Y., Hanratty B., Ang L.S., Bhatia V. (2023). Reversible Epigenetic Alterations Mediate PSMA Expression Heterogeneity in Advanced Metastatic Prostate Cancer. JCI Insight.

[17] Bakht M.K., Beltran H. (2024). Aiming High: Prostate-Specific Membrane Antigen Expression in Treatment-Naïve Prostate Cancer. Eur. Urol..

[18] Wyatt A.W., Annala M., Aggarwal R., Beja K., Feng F., Youngren J., Foye A., Lloyd P., Nykter M., Beer T.M. (2017). Concordance of Circulating Tumor DNA and Matched Metastatic Tissue Biopsy in Prostate Cancer. J. Natl. Cancer Inst..

[19] Ried K., Tamanna T., Matthews S., Eng P., Sali A. (2020). New Screening Test Improves Detection of Prostate Cancer Using Circulating Tumor Cells and Prostate-Specific Markers. Front. Oncol..

[20] McKiernan J., Donovan M.J., O’Neill V., Bentink S., Noerholm M., Belzer S., Skog J., Kattan M.W., Partin A., Andriole G. (2016). A Novel Urine Exosome Gene Expression Assay to Predict High-Grade Prostate Cancer at Initial Biopsy. JAMA Oncol..

[21] Ramirez-Garrastacho M., Bajo-Santos C., Line A., Martens-Uzunova E.S., de la Fuente J.M., Moros M., Soekmadji C., Tasken K.A., Llorente A. (2022). Extracellular Vesicles as a Source of Prostate Cancer Biomarkers in Liquid Biopsies: A Decade of Research. Br. J. Cancer.

[22] Wang C.-B., Chen S.-H., Zhao L., Jin X., Chen X., Ji J., Mo Z.-N., Wang F.-B. (2023). Urine-Derived Exosomal PSMA Is a Promising Diagnostic Biomarker for the Detection of Prostate Cancer on Initial Biopsy. Clin. Transl. Oncol..

[23] Matijašević Joković S., Korać A., Kovačević S., Djordjević A., Filipović L., Dobrijević Z., Brkušanin M., Savić-Pavićević D., Vuković I., Popović M. (2024). Exosomal Prostate-Specific Membrane Antigen (PSMA) and Caveolin-1 as Potential Biomarkers of Prostate Cancer—Evidence from Serbian Population. Int. J. Mol. Sci..

[24] Gupta S., Fernandez L., Bourdon D., Hamid A.A., Pasam A., Lam E., Wenstrup R., Sandhu S. (2024). Detection of PSMA Expression on Circulating Tumor Cells by Blood-Based Liquid Biopsy in Prostate Cancer. J. Circ. Biomark..

[25] Hamid Y., Rabbani R.D., Afsara R., Nowrin S., Ghose A., Papadopoulos V., Sirlantzis K., Ovsepian S.V., Boussios S. (2025). Exosomal Liquid Biopsy in Prostate Cancer: A Systematic Review of Biomarkers for Diagnosis, Prognosis, and Treatment Response. Int. J. Mol. Sci..

[26] Jochumsen M.R., Bouchelouche K. (2024). PSMA PET/CT for Primary Staging of Prostate Cancer—An Updated Overview. Semin. Nucl. Med..

[27] von Stauffenberg F., Poyet C., Beintner-Skawran S., Maurer A., Schmid F.A. (2024). Current Clinical Applications of PSMA-PET for Prostate Cancer Diagnosis, Staging, and Treatment. Cancers.

[28] Lamb H.M., Faulds D. (1998). Capromab Pendetide. A Review of Its Use as an Imaging Agent in Prostate Cancer. Drugs Aging.

[29] Holland J.P., Divilov V., Bander N.H., Smith-Jones P.M., Larson S.M., Lewis J.S. (2010). 89Zr-DFO-J591 for immunoPET of Prostate-Specific Membrane Antigen Expression In Vivo. J. Nucl. Med..

[30] Pandit-Taskar N., O’Donoghue J.A., Ruan S., Lyashchenko S.K., Carrasquillo J.A., Heller G., Martinez D.F., Cheal S.M., Lewis J.S., Fleisher M. (2016). First-in-Human Imaging with 89Zr-Df-IAB2M Anti-PSMA Minibody in Patients with Metastatic Prostate Cancer: Pharmacokinetics, Biodistribution, Dosimetry, and Lesion Uptake. J. Nucl. Med..

[31] Evazalipour M., D’Huyvetter M., Tehrani B.S., Abolhassani M., Omidfar K., Abdoli S., Arezumand R., Morovvati H., Lahoutte T., Muyldermans S. (2014). Generation and Characterization of Nanobodies Targeting PSMA for Molecular Imaging of Prostate Cancer. Contrast Media Mol. Imaging.

[32] Chatalic K.L.S., Veldhoven-Zweistra J., Bolkestein M., Hoeben S., Koning G.A., Boerman O.C., de Jong M., van Weerden W.M. (2015). A Novel 111In-Labeled Anti–Prostate-Specific Membrane Antigen Nanobody for Targeted SPECT/CT Imaging of Prostate Cancer. J. Nucl. Med..

[33] Fan X., Wang L., Guo Y., Tu Z., Li L., Tong H., Xu Y., Li R., Fang K. (2015). Ultrasonic Nanobubbles Carrying Anti-PSMA Nanobody: Construction and Application in Prostate Cancer-Targeted Imaging. PLoS ONE.

[34] Emmett L.M., Papa N., Buteau J., Ho B., Liu V., Roberts M., Thompson J., Moon D., Sheehan-Dare G., Alghazo O. (2022). The PRIMARY Score: Using Intra-Prostatic PSMA PET/CT Patterns to Optimise Prostate Cancer Diagnosis. J. Nucl. Med..

[35] Sonni I., Felker E.R., Lenis A.T., Sisk A.E., Bahri S., Allen-Auerbach M., Armstrong W.R., Suvannarerg V., Tubtawee T., Grogan T. (2022). Head-to-Head Comparison of 68Ga-PSMA-11 PET/CT and mpMRI with a Histopathology Gold Standard in the Detection, Intraprostatic Localization, and Determination of Local Extension of Primary Prostate Cancer: Results from a Prospective Single-Center Imaging Trial. J. Nucl. Med..

[36] Schmuck S., Mamach M., Wilke F., von Klot C.A., Henkenberens C., Thackeray J.T., Sohns J.M., Geworski L., Ross T.L., Wester H.-J. (2017). Multiple Time-Point 68Ga-PSMA I&T PET/CT for Characterization of Primary Prostate Cancer: Value of Early Dynamic and Delayed Imaging. Clin. Nucl. Med..

[37] Cytawa W., Seitz A.K., Kircher S., Fukushima K., Tran-Gia J., Schirbel A., Bandurski T., Lass P., Krebs M., Połom W. (2020). 68Ga-PSMA I&T PET/CT for Primary Staging of Prostate Cancer. Eur. J. Nucl. Med. Mol. Imaging.

[38] McCarthy M., Langton T., Kumar D., Campbell A. (2017). Comparison of PSMA-HBED and PSMA-I&T as Diagnostic Agents in Prostate Carcinoma. Eur. J. Nucl. Med. Mol. Imaging.

[39] Rowe S.P., Gage K.L., Faraj S.F., Macura K.J., Cornish T.C., Gonzalez-Roibon N., Guner G., Munari E., Partin A.W., Pavlovich C.P. (2015). 18F-DCFBC PET/CT for PSMA-Based Detection and Characterization of Primary Prostate Cancer. J. Nucl. Med..

[40] Turkbey B., Mena E., Lindenberg L., Adler S., Bednarova S., Berman R., Ton A.T., McKinney Y., Eclarinal P., Hill C. (2017). 18F-DCFBC Prostate-Specific Membrane Antigen-Targeted PET/CT Imaging in Localized Prostate Cancer: Correlation with Multiparametric MRI and Histopathology. Clin. Nucl. Med..

[41] Chen Y., Pullambhatla M., Foss C.A., Byun Y., Nimmagadda S., Senthamizhchelvan S., Sgouros G., Mease R.C., Pomper M.G. (2011). 2-(3-{1-Carboxy-5-[(6-[18F]Fluoro-Pyridine-3-Carbonyl)-Amino]-Pentyl}-Ureido)-Pentanedioic Acid, [18F]DCFPyL, a PSMA-Based PET Imaging Agent for Prostate Cancer. Clin. Cancer Res..

[42] Zhou S., Liu T., Zhu Z., Zhang L., Qian S., Fu H., Cao Q., Kang J. (2022). 18F-DCFPyL PET/CT in Newly Diagnosed Prostate Cancer: Diagnostic Value of Intraprostatic PSMA Uptake in Risk Classification of Prostate Cancer. Front. Oncol..

[43] Giesel F.L., Hadaschik B., Cardinale J., Radtke J., Vinsensia M., Lehnert W., Kesch C., Tolstov Y., Singer S., Grabe N. (2017). F-18 Labelled PSMA-1007: Biodistribution, Radiation Dosimetry and Histopathological Validation of Tumor Lesions in Prostate Cancer Patients. Eur. J. Nucl. Med. Mol. Imaging.

[44] Ye Z., Kou Y., Shen J., Dang J., Tan X., Jiang X., Wang X., Lu H., Chen S., Cheng Z. (2024). A Comparative Study of 18F-PSMA-1007 PET/CT and Pelvic MRI in Newly Diagnosed Prostate Cancer. BMC Med. Imaging.

[45] Mookerji N., Pfanner T., Hui A., Huang G., Albers P., Mittal R., Broomfield S., Dean L., St Martin B., Jacobsen N.-E. (2024). Fluorine-18 Prostate-Specific Membrane Antigen-1007 PET/CT vs Multiparametric MRI for Locoregional Staging of Prostate Cancer. JAMA Oncol..

[46] Pattison D.A., Debowski M., Gulhane B., Arnfield E.G., Pelecanos A.M., Garcia P.L., Latter M.J., Lin C.Y., Roberts M.J., Ramsay S.C. (2022). Prospective Intra-Individual Blinded Comparison of [18F]PSMA-1007 and [68 Ga]Ga-PSMA-11 PET/CT Imaging in Patients with Confirmed Prostate Cancer. Eur. J. Nucl. Med. Mol. Imaging.

[47] Rauscher I., Krönke M., König M., Gafita A., Maurer T., Horn T., Schiller K., Weber W., Eiber M. (2020). Matched-Pair Comparison of 68Ga-PSMA-11 PET/CT and 18F-PSMA-1007 PET/CT: Frequency of Pitfalls and Detection Efficacy in Biochemical Recurrence After Radical Prostatectomy. J. Nucl. Med..

[48] Pepe P., Pennisi M. (2023). Targeted Biopsy in Men High Risk for Prostate Cancer: 68Ga-PSMA PET/CT Versus mpMRI. Clin. Genitourin. Cancer.

[49] Pepe P., Pepe L., Cosentino S., Ippolito M., Pennisi M., Fraggetta F. (2022). Detection Rate of 68Ga-PSMA PET/CT vs. mpMRI Targeted Biopsy for Clinically Significant Prostate Cancer. Anticancer Res..

[50] Checcucci E., Bauckneht M., Cisero E., Volpi G., Rizzo A., Zattoni F., Bianchi L., De Angelis M., Cangemi D., Heetman J. (2025). PSMA PET-Targeted Biopsy for Prostate Cancer Diagnosis: Initial Experience from a Multicenter Cohort. Urology.

[51] Bianchi L., Cangemi D., Farolfi A., Sgro C.M.P., Giorgio A.D., Castellucci P., Gaudiano C., Corcioni B., Giunchi F., Degiovanni A. (2025). PSMA-Targeted Biopsy with Fusion Guidance for Detecting Clinically Significant Prostate Cancer in Men with Negative MRI-Feasibility and Diagnostic Performance of a Pilot Single-Center Prospective Study. Clin. Genitourin. Cancer.

[52] Chow K.M., Lee A., Peh D., Tan Y.G., Tay K.J., Ho H., Cheng C., Lam W., Thang S.P., Tuan J. (2025). Combined Prostate-Specific Membrane Antigen Positron Emission Tomography and Multiparametric Magnetic Resonance Imaging for the Diagnosis of Clinically Significant Prostate Cancer. Eur. Urol. Oncol..

[53] Mazzone E., Cannoletta D., Quarta L., Chen D.C., Thomson A., Barletta F., Stabile A., Moon D., Eapen R., Lawrentschuk N. (2025). A Comprehensive Systematic Review and Meta-Analysis of the Role of Prostate-Specific Membrane Antigen Positron Emission Tomography for Prostate Cancer Diagnosis and Primary Staging before Definitive Treatment. Eur. Urol..

[54] Petersen L.J., Zacho H.D. (2020). PSMA PET for Primary Lymph Node Staging of Intermediate and High-Risk Prostate Cancer: An Expedited Systematic Review. Cancer Imaging.

[55] Stabile A., Pellegrino A., Mazzone E., Cannoletta D., de Angelis M., Barletta F., Scuderi S., Cucchiara V., Gandaglia G., Raggi D. (2022). Can Negative Prostate-Specific Membrane Antigen Positron Emission Tomography/Computed Tomography Avoid the Need for Pelvic Lymph Node Dissection in Newly Diagnosed Prostate Cancer Patients? A Systematic Review and Meta-Analysis with Backup Histology as Reference Standard. Eur. Urol. Oncol..

[56] Hope T.A., Eiber M., Armstrong W.R., Juarez R., Murthy V., Lawhn-Heath C., Behr S.C., Zhang L., Barbato F., Ceci F. (2021). Diagnostic Accuracy of 68Ga-PSMA-11 PET for Pelvic Nodal Metastasis Detection Prior to Radical Prostatectomy and Pelvic Lymph Node Dissection: A Multicenter Prospective Phase 3 Imaging Trial. JAMA Oncol..

[57] Rajwa P., Heidenreich J., Drzezga A., Schmidt M., Shariat S.F., Heidenreich A. (2024). The Diagnostic Accuracy of 68 Ga-PSMA-PET/CT in Primary Staging of Patients with High-Risk Nonmetastatic Prostate Cancer Treated with Radical Prostatectomy: A Single-Center Cohort Analysis. Prostate.

[58] Ingvar J., Hvittfeldt E., Trägårdh E., Simoulis A., Bjartell A. (2022). Assessing the Accuracy of [18F]PSMA-1007 PET/CT for Primary Staging of Lymph Node Metastases in Intermediate- and High-Risk Prostate Cancer Patients. EJNMMI Res..

[59] Jansen B.H.E., Bodar Y.J.L., Zwezerijnen G.J.C., Meijer D., van der Voorn J.P., Nieuwenhuijzen J.A., Wondergem M., Roeleveld T.A., Boellaard R., Hoekstra O.S. (2021). Pelvic Lymph-Node Staging with 18F-DCFPyL PET/CT Prior to Extended Pelvic Lymph-Node Dissection in Primary Prostate Cancer—The SALT Trial. Eur. J. Nucl. Med. Mol. Imaging.

[60] Pienta K.J., Gorin M.A., Rowe S.P., Carroll P.R., Pouliot F., Probst S., Saperstein L., Preston M.A., Alva A.S., Patnaik A. (2021). A Phase 2/3 Prospective Multicenter Study of the Diagnostic Accuracy of Prostate Specific Membrane Antigen PET/CT with 18F-DCFPyL in Prostate Cancer Patients (OSPREY). J. Urol..

[61] Cookson M.S., Aus G., Burnett A.L., Canby-Hagino E.D., D’Amico A.V., Dmochowski R.R., Eton D.T., Forman J.D., Goldenberg S.L., Hernandez J. (2007). Variation in the Definition of Biochemical Recurrence in Patients Treated for Localized Prostate Cancer: The American Urological Association Prostate Guidelines for Localized Prostate Cancer Update Panel Report and Recommendations for a Standard in the Reporting of Surgical Outcomes. J. Urol..

[62] Matsukawa A., Yanagisawa T., Fazekas T., Miszczyk M., Tsuboi I., Kardoust Parizi M., Laukhtina E., Klemm J., Mancon S., Mori K. (2025). Salvage Therapies for Biochemical Recurrence after Definitive Local Treatment: A Systematic Review, Meta-Analysis, and Network Meta-Analysis. Prostate Cancer Prostatic Dis..

[63] Roach M., Hanks G., Thames H., Schellhammer P., Shipley W.U., Sokol G.H., Sandler H. (2006). Defining Biochemical Failure Following Radiotherapy with or without Hormonal Therapy in Men with Clinically Localized Prostate Cancer: Recommendations of the RTOG-ASTRO Phoenix Consensus Conference. Int. J. Radiat. Oncol. Biol. Phys..

[64] Hofman M.S., Lawrentschuk N., Francis R.J., Tang C., Vela I., Thomas P., Rutherford N., Martin J.M., Frydenberg M., Shakher R. (2020). Prostate-Specific Membrane Antigen PET-CT in Patients with High-Risk Prostate Cancer before Curative-Intent Surgery or Radiotherapy (proPSMA): A Prospective, Randomised, Multicentre Study. Lancet.

[65] Fendler W.P., Calais J., Eiber M., Flavell R.R., Mishoe A., Feng F.Y., Nguyen H.G., Reiter R.E., Rettig M.B., Okamoto S. (2019). Assessment of 68Ga-PSMA-11 PET Accuracy in Localizing Recurrent Prostate Cancer: A Prospective Single-Arm Clinical Trial. JAMA Oncol..

[66] Morgan T.M., Boorjian S.A., Buyyounouski M.K., Chapin B.F., Chen D.Y.T., Cheng H.H., Chou R., Jacene H.A., Kamran S.C., Kim S.K. (2024). Salvage Therapy for Prostate Cancer: AUA/ASTRO/SUO Guideline Part I: Introduction and Treatment Decision-Making at the Time of Suspected Biochemical Recurrence after Radical Prostatectomy. J. Urol..

[67] Morris M.J., Rowe S.P., Gorin M.A., Saperstein L., Pouliot F., Josephson D., Wong J.Y.C., Pantel A.R., Cho S.Y., Gage K.L. (2021). Diagnostic Performance of 18F-DCFPyL-PET/CT in Men with Biochemically Recurrent Prostate Cancer: Results from the CONDOR Phase III, Multicenter Study. Clin. Cancer Res..

[68] Laudicella R., La Torre F., Davì V., Crocè L., Aricò D., Leonardi G., Russo S., Minutoli F., Burger I.A., Baldari S. (2022). Prostate Cancer Biochemical Recurrence Resulted Negative on [68Ga]Ga-PSMA-11 but Positive on [18F]Fluoromethylcholine PET/CT. Tomography.

[69] Crocerossa F., Marchioni M., Novara G., Carbonara U., Ferro M., Russo G.I., Porpiglia F., Nicola M.D., Damiano R., Autorino R. (2021). Detection Rate of Prostate Specific Membrane Antigen Tracers for Positron Emission Tomography/Computerized Tomography in Prostate Cancer Biochemical Recurrence: A Systematic Review and Network Meta-Analysis. J. Urol..

[70] Yilmaz U., Komek H., Can C., Altindag S. (2019). The Role of (68Ga)PSMA I&T in Biochemical Recurrence after Radical Prostatectomy: Detection Rate and the Correlation between the Level of PSA, Gleason Score, and the SUVmax. Ann. Nucl. Med..

[71] Mena E., Lindenberg M.L., Shih J.H., Adler S., Harmon S., Bergvall E., Citrin D., Dahut W., Ton A.T., McKinney Y. (2018). Clinical Impact of PSMA-Based 18F-DCFBC PET/CT Imaging in Patients with Biochemically Recurrent Prostate Cancer after Primary Local Therapy. Eur. J. Nucl. Med. Mol. Imaging.

[72] Belliveau C., Saad F., Duplan D., Petit C., Delouya G., Taussky D., Barkati M., Lambert C., Beauchemin M.-C., Clavel S. (2025). Prostate-Specific Membrane Antigen PET-Guided Intensification of Salvage Radiotherapy After Radical Prostatectomy: A Phase 2 Randomized Clinical Trial. JAMA Oncol..

[73] Kleiburg F., de Geus-Oei L.F., Luelmo S.a.C., Spijkerman R., Goeman J.J., Toonen F.a.J., Smit F., van der Hulle T., Heijmen L. (2024). PSMA PET/CT for Treatment Response Evaluation at Predefined Time Points Is Superior to PSA Response for Predicting Survival in Metastatic Castration-Resistant Prostate Cancer Patients. Eur. J. Radiol..

[74] Gafita A., Djaileb L., Rauscher I., Fendler W.P., Hadaschik B., Rowe S.P., Herrmann K., Solnes L.B., Calais J., Rettig M.B. (2024). RECIP 1.0 Predicts Progression-Free Survival After [177Lu]Lu-PSMA Radiopharmaceutical Therapy in Patients with Metastatic Castration-Resistant Prostate Cancer. J. Nucl. Med..

[75] Gafita A., Djaileb L., Rauscher I., Fendler W.P., Hadaschik B., Rowe S.P., Herrmann K., Calais J., Rettig M., Eiber M. (2023). Response Evaluation Criteria in PSMA PET/CT (RECIP 1.0) in Metastatic Castration-Resistant Prostate Cancer. Radiology.

[76] Shagera Q.A., Karfis I., Kristanto P., Spyridon S., Diamand R., Santapau A., Peltier A., Roumeguère T., Flamen P., Artigas C. (2023). PSMA PET/CT for Response Assessment and Overall Survival Prediction in Patients with Metastatic Castration-Resistant Prostate Cancer Treated with Androgen Receptor Pathway Inhibitors. J. Nucl. Med..

[77] Nguyen H.G., van den Berg N.S., Antaris A.L., Xue L., Greenberg S., Rosenthal J.W., Muchnik A., Klaassen A., Simko J.P., Dutta S. (2024). First-in-Human Evaluation of a Prostate-Specific Membrane Antigen-Targeted Near-Infrared Fluorescent Small Molecule for Fluorescence-Based Identification of Prostate Cancer in Patients with High-Risk Prostate Cancer Undergoing Robotic-Assisted Prostatectomy. Eur. Urol. Oncol..

[78] Stibbe J.A., de Barros H.A., Linders D.G.J., Bhairosingh S.S., Bekers E.M., van Leeuwen P.J., Low P.S., Kularatne S.A., Vahrmeijer A.L., Burggraaf J. (2023). First-in-Patient Study of OTL78 for Intraoperative Fluorescence Imaging of Prostate-Specific Membrane Antigen-Positive Prostate Cancer: A Single-Arm, Phase 2a, Feasibility Trial. Lancet Oncol..

[79] Hamdy F.C., Lamb A.D., Tullis I.D.C., Verrill C., Rombach I., Rao S.R., Colling R., Barber P.R., Volpi D., Barbera-Martin L. (2024). First-in-Man Study of the PSMA Minibody IR800-IAB2M for Molecularly Targeted Intraoperative Fluorescence Guidance during Radical Prostatectomy. Eur. J. Nucl. Med. Mol. Imaging.

[80] Banerjee S.R., Pullambhatla M., Byun Y., Nimmagadda S., Foss C.A., Green G., Fox J.J., Lupold S.E., Mease R.C., Pomper M.G. (2011). Sequential SPECT and Optical Imaging of Experimental Models of Prostate Cancer with a Dual Modality Inhibitor of the Prostate-Specific Membrane Antigen. Angew. Chem. Int. Ed. Engl..

[81] Lütje S., Rijpkema M., Franssen G.M., Fracasso G., Helfrich W., Eek A., Oyen W.J., Colombatti M., Boerman O.C. (2014). Dual-Modality Image-Guided Surgery of Prostate Cancer with a Radiolabeled Fluorescent Anti-PSMA Monoclonal Antibody. J. Nucl. Med..

[82] Schottelius M., Wurzer A., Wissmiller K., Beck R., Koch M., Gorpas D., Notni J., Buckle T., van Oosterom M.N., Steiger K. (2019). Synthesis and Preclinical Characterization of the PSMA-Targeted Hybrid Tracer PSMA-I&F for Nuclear and Fluorescence Imaging of Prostate Cancer. J. Nucl. Med..

[83] Eder A.-C., Matthias J., Schäfer M., Schmidt J., Steinacker N., Bauder-Wüst U., Domogalla L.-C., Roscher M., Haberkorn U., Eder M. (2022). A New Class of PSMA-617-Based Hybrid Molecules for Preoperative Imaging and Intraoperative Fluorescence Navigation of Prostate Cancer. Pharmaceuticals.

[84] Li Y., Duan X., Xu H., Zhang J., Zhou H., Zhang X., Zhang J., Yang Z., Hu Z., Zhang N. (2022). Optimization of ODAP-Urea-Based Dual-Modality PSMA Targeting Probes for Sequential PET-CT and Optical Imaging. Bioorg Med. Chem..

[85] Chen S., Xu H., Chen X., Shen Q., Chen X., Zhang M., Li Z., Zhang Z., Hao H., Yu W. (2025). First-in-Human Study of a Dual-Modality Prostate-Specific Membrane Antigen-Targeted Probe for Preoperative Positron Emission Tomography/Computed Tomography Imaging and Intraoperative Fluorescence Imaging in Prostate Cancer. Eur. Urol..

[86] Jiang Z., Kadeerhan G., Zhang J., Guo W., Guo H., Wang D. (2025). Advances in Prostate-Specific Membrane Antigen-Targeted Theranostics: From Radionuclides to near-Infrared Fluorescence Technology. Front. Immunol..

[87] Berrens A.-C., Knipper S., Marra G., van Leeuwen P.J., van der Mierden S., Donswijk M.L., Maurer T., van Leeuwen F.W.B., van der Poel H.G. (2023). State of the Art in Prostate-Specific Membrane Antigen-Targeted Surgery—A Systematic Review. Eur. Urol. Open Sci..

[88] Derks Y.H.W., Löwik D.W.P.M., Sedelaar J.P.M., Gotthardt M., Boerman O.C., Rijpkema M., Lütje S., Heskamp S. (2019). PSMA-Targeting Agents for Radio- and Fluorescence-Guided Prostate Cancer Surgery. Theranostics.

[89] Carter R.E., Feldman A.R., Coyle J.T. (1996). Prostate-Specific Membrane Antigen Is a Hydrolase with Substrate and Pharmacologic Characteristics of a Neuropeptidase. Proc. Natl. Acad. Sci. USA.

[90] Kaittanis C., Andreou C., Hieronymus H., Mao N., Foss C.A., Eiber M., Weirich G., Panchal P., Gopalan A., Zurita J. (2018). Prostate-Specific Membrane Antigen Cleavage of Vitamin B9 Stimulates Oncogenic Signaling through Metabotropic Glutamate Receptors. J. Exp. Med..

[91] Evans M.J., Smith-Jones P.M., Wongvipat J., Navarro V., Kim S., Bander N.H., Larson S.M., Sawyers C.L. (2011). Noninvasive Measurement of Androgen Receptor Signaling with a Positron-Emitting Radiopharmaceutical That Targets Prostate-Specific Membrane Antigen. Proc. Natl. Acad. Sci. USA.

[92] Perner S., Hofer M.D., Kim R., Shah R.B., Li H., Möller P., Hautmann R.E., Gschwend J.E., Kuefer R., Rubin M.A. (2007). Prostate-Specific Membrane Antigen Expression as a Predictor of Prostate Cancer Progression. Hum. Pathol..

[93] Sartor O., de Bono J., Chi K.N., Fizazi K., Herrmann K., Rahbar K., Tagawa S.T., Nordquist L.T., Vaishampayan N., El-Haddad G. (2021). Lutetium-177-PSMA-617 for Metastatic Castration-Resistant Prostate Cancer. N. Engl. J. Med..

[94] Morris M.J., Castellano D., Herrmann K., de Bono J.S., Shore N.D., Chi K.N., Crosby M., Piulats J.M., Fléchon A., Wei X.X. (2024). 177Lu-PSMA-617 versus a Change of Androgen Receptor Pathway Inhibitor Therapy for Taxane-Naive Patients with Progressive Metastatic Castration-Resistant Prostate Cancer (PSMAfore): A Phase 3, Randomised, Controlled Trial. Lancet.

[95] Hofman M.S., Emmett L., Sandhu S., Iravani A., Joshua A.M., Goh J.C., Pattison D.A., Tan T.H., Kirkwood I.D., Ng S. (2021). [177Lu]Lu-PSMA-617 versus Cabazitaxel in Patients with Metastatic Castration-Resistant Prostate Cancer (TheraP): A Randomised, Open-Label, Phase 2 Trial. Lancet.

[96] Gafita A., Martin A.J., Emmett L., Eiber M., Iravani A., Fendler W.P., Buteau J., Sandhu S., Azad A.A., Herrmann K. (2025). Validation of Prognostic and Predictive Models for Therapeutic Response in Patients Treated with [177Lu]Lu-PSMA-617 Versus Cabazitaxel for Metastatic Castration-Resistant Prostate Cancer (TheraP): A Post Hoc Analysis from a Randomised, Open-Label, Phase 2 Trial. Eur. Urol. Oncol..

[97] Flippot R., Telli T., Velev M., Fléchon A., De Vries-Brilland M., Turpin L., Bergman A., Turco F., Mahammedi H., Fendler W.P. (2024). Activity of Lutetium-177 Prostate-Specific Membrane Antigen and Determinants of Outcomes in Patients with Metastatic Castration-Resistant Prostate Cancer Previously Treated with Cabazitaxel: The PACAP Study. Eur. Urol. Oncol..

[98] Azad A.A., Bressel M., Tan H., Voskoboynik M., Suder A., Weickhardt A.J., Guminski A., Francis R.J., Saghebi J., Dhiantravan N. (2024). Sequential [177Lu]Lu-PSMA-617 and Docetaxel versus Docetaxel in Patients with Metastatic Hormone-Sensitive Prostate Cancer (UpFrontPSMA): A Multicentre, Open-Label, Randomised, Phase 2 Study. Lancet Oncol..

[99] Sathekge M., Bruchertseifer F., Knoesen O., Reyneke F., Lawal I., Lengana T., Davis C., Mahapane J., Corbett C., Vorster M. (2019). 225Ac-PSMA-617 in Chemotherapy-Naive Patients with Advanced Prostate Cancer: A Pilot Study. Eur. J. Nucl. Med. Mol. Imaging.

[100] Sathekge M., Bruchertseifer F., Vorster M., Lawal I., Knoesen O., Mahapane J., Davis C., Mdlophane A., Maes A., Mokoala K. (2022). mCRPC Patients Receiving 225Ac-PSMA-617 Therapy in Post Androgen Deprivation Therapy Setting: Response to Treatment and Survival Analysis. J. Nucl. Med..

[101] Sathekge M.M., Lawal I.O., Bal C., Bruchertseifer F., Ballal S., Cardaci G., Davis C., Eiber M., Hekimsoy T., Knoesen O. (2024). Actinium-225-PSMA Radioligand Therapy of Metastatic Castration-Resistant Prostate Cancer (WARMTH Act): A Multicentre, Retrospective Study. Lancet Oncol..

[102] Feuerecker B., Tauber R., Knorr K., Heck M., Beheshti A., Seidl C., Bruchertseifer F., Pickhard A., Gafita A., Kratochwil C. (2021). Activity and Adverse Events of Actinium-225-PSMA-617 in Advanced Metastatic Castration-Resistant Prostate Cancer After Failure of Lutetium-177-PSMA. Eur. Urol..

[103] Yadav M.P., Ballal S., Sahoo R.K., Tripathi M., Seth A., Bal C. (2020). Efficacy and Safety of 225Ac-PSMA-617 Targeted Alpha Therapy in Metastatic Castration-Resistant Prostate Cancer Patients. Theranostics.

[104] Rathke H., Winter E., Bruchertseifer F., Röhrich M., Giesel F.L., Haberkorn U., Morgenstern A., Kratochwil C. (2024). Deescalated 225Ac-PSMA-617 Versus 177Lu/225Ac-PSMA-617 Cocktail Therapy: A Single-Center Retrospective Analysis of 233 Patients. J. Nucl. Med..

[105] Khreish F., Ebert N., Ries M., Maus S., Rosar F., Bohnenberger H., Stemler T., Saar M., Bartholomä M., Ezziddin S. (2020). 225Ac-PSMA-617/177Lu-PSMA-617 Tandem Therapy of Metastatic Castration-Resistant Prostate Cancer: Pilot Experience. Eur. J. Nucl. Med. Mol. Imaging.

[106] Groener D., Nguyen C.T., Baumgarten J., Bockisch B., Davis K., Happel C., Mader N., Nguyen Ngoc C., Wichert J., Banek S. (2021). Hematologic Safety of 177Lu-PSMA-617 Radioligand Therapy in Patients with Metastatic Castration-Resistant Prostate Cancer. EJNMMI Res..

[107] Barber T.W., Singh A., Kulkarni H.R., Niepsch K., Billah B., Baum R.P. (2019). Clinical Outcomes of 177Lu-PSMA Radioligand Therapy in Earlier and Later Phases of Metastatic Castration-Resistant Prostate Cancer Grouped by Previous Taxane Chemotherapy. J. Nucl. Med..

[108] Herrmann K., Rahbar K., Eiber M., Sparks R., Baca N., Krause B.J., Lassmann M., Jentzen W., Tang J., Chicco D. (2024). Renal and Multiorgan Safety of 177Lu-PSMA-617 in Patients with Metastatic Castration-Resistant Prostate Cancer in the VISION Dosimetry Substudy. J. Nucl. Med..

[109] Gallyamov M., Meyrick D., Barley J., Lenzo N. (2020). Renal Outcomes of Radioligand Therapy: Experience of 177lutetium-Prostate-Specific Membrane Antigen Ligand Therapy in Metastatic Castrate-Resistant Prostate Cancer. Clin. Kidney J..

[110] Rivero-Buceta E., Vidaurre-Agut C., Vera-Donoso C.D., Benlloch J.M., Moreno-Manzano V., Botella P. (2019). PSMA-Targeted Mesoporous Silica Nanoparticles for Selective Intracellular Delivery of Docetaxel in Prostate Cancer Cells. ACS Omega.

[111] Mangadlao J.D., Wang X., McCleese C., Escamilla M., Ramamurthy G., Wang Z., Govande M., Basilion J.P., Burda C. (2018). Prostate-Specific Membrane Antigen Targeted Gold Nanoparticles for Theranostics of Prostate Cancer. ACS Nano.

[112] Dai L., Shen G., Wang Y., Yang P., Wang H., Liu Z. (2021). PSMA-Targeted Melanin-like Nanoparticles as a Multifunctional Nanoplatform for Prostate Cancer Theranostics. J. Mater. Chem. B.

[113] Chen Z., He Z., Li X., Wei Y., Xu H., Lin Y., Wei X., Huang Y., Hou J., Wang H. (2025). NIR-II Imaging-Guided Self-Enhanced Nanomedicine with Reactive Oxygen Species Amplification for Type I Photodynamic Therapy of Prostate Cancer. Adv. Funct. Mater..

[114] Adekiya T.A., Hudson T., Bakare O., Ameyaw E.E., Adebayo A., Olajubutu O., Adesina S.K. (2024). PSMA-Targeted Combination Brusatol and Docetaxel Nanotherapeutics for the Treatment of Prostate Cancer. Biomed. Pharmacother..

[115] Afsharzadeh M., Hashemi M., Babaei M., Abnous K., Ramezani M. (2020). PEG-PLA Nanoparticles Decorated with Small-Molecule PSMA Ligand for Targeted Delivery of Galbanic Acid and Docetaxel to Prostate Cancer Cells. J. Cell. Physiol..

[116] Yin L., Yang F., Wang W., Zhang L., Cao Z., Shi H., Pan K., Wu L., Xiao H., Xing N. (2025). PSMA-Targeted Nanoparticles with PI3K/mTOR Dual Inhibitor Downregulate P-Glycoprotein and Inactivate Myeloid-Derived Suppressor Cells for Enhanced Chemotherapy and Immunotherapy in Prostate Cancer. Adv. Mater..

[117] Von Hoff D.D., Mita M.M., Ramanathan R.K., Weiss G.J., Mita A.C., LoRusso P.M., Burris H.A., Hart L.L., Low S.C., Parsons D.M. (2016). Phase I Study of PSMA-Targeted Docetaxel-Containing Nanoparticle BIND-014 in Patients with Advanced Solid Tumors. Clin. Cancer Res..

[118] Autio K.A., Dreicer R., Anderson J., Garcia J.A., Alva A., Hart L.L., Milowsky M.I., Posadas E.M., Ryan C.J., Graf R.P. (2018). Safety and Efficacy of BIND-014, a Docetaxel Nanoparticle Targeting Prostate-Specific Membrane Antigen for Patients with Metastatic Castration-Resistant Prostate Cancer: A Phase 2 Clinical Trial. JAMA Oncol..

[119] Henry M.D., Wen S., Silva M.D., Chandra S., Milton M., Worland P.J. (2004). A Prostate-Specific Membrane Antigen-Targeted Monoclonal Antibody–Chemotherapeutic Conjugate Designed for the Treatment of Prostate Cancer. Cancer Res..

[120] Galsky M.D., Eisenberger M., Moore-Cooper S., Kelly W.K., Slovin S.F., DeLaCruz A., Lee Y., Webb I.J., Scher H.I. (2008). Phase I Trial of the Prostate-Specific Membrane Antigen-Directed Immunoconjugate MLN2704 in Patients with Progressive Metastatic Castration-Resistant Prostate Cancer. J. Clin. Oncol..

[121] Milowsky M.I., Galsky M.D., Morris M.J., Crona D.J., George D.J., Dreicer R., Tse K., Petruck J., Webb I.J., Bander N.H. (2016). Phase 1/2 Multiple Ascending Dose Trial of the Prostate-Specific Membrane Antigen-Targeted Antibody Drug Conjugate MLN2704 in Metastatic Castration-Resistant Prostate Cancer. Urol. Oncol. Semin. Orig. Investig..

[122] Petrylak D.P., Kantoff P., Vogelzang N.J., Mega A., Fleming M.T., Stephenson J.J., Frank R., Shore N.D., Dreicer R., McClay E.F. (2019). Phase 1 Study of PSMA ADC, an Antibody-drug Conjugate Targeting Prostate-specific Membrane Antigen, in Chemotherapy-refractory Prostate Cancer. Prostate.

[123] Petrylak D.P., Vogelzang N.J., Chatta K., Fleming M.T., Smith D.C., Appleman L.J., Hussain A., Modiano M., Singh P., Tagawa S.T. (2020). PSMA ADC Monotherapy in Patients with Progressive Metastatic Castration-Resistant Prostate Cancer Following Abiraterone and/or Enzalutamide: Efficacy and Safety in Open-Label Single-Arm Phase 2 Study. Prostate.

[124] Cho S., Zammarchi F., Williams D.G., Havenith C.E.G., Monks N.R., Tyrer P., D’Hooge F., Fleming R., Vashisht K., Dimasi N. (2018). Antitumor Activity of MEDI3726 (ADCT-401), a Pyrrolobenzodiazepine Antibody–Drug Conjugate Targeting PSMA, in Preclinical Models of Prostate Cancer. Mol. Cancer Ther..

[125] de Bono J.S., Fleming M.T., Wang J.S., Cathomas R., Miralles M.S., Bothos J., Hinrichs M.J., Zhang Q., He P., Williams M. (2021). Phase I Study of MEDI3726: A Prostate-Specific Membrane Antigen-Targeted Antibody–Drug Conjugate, in Patients with mCRPC after Failure of Abiraterone or Enzalutamide. Clin. Cancer Res..

[126] Skidmore L.K., Mills D., Kim J.Y., Knudsen N.A., Nelson J.D., Pal M., Wang J., GC K., Gray M.J., Barkho W. (2024). Preclinical Characterization of ARX517, a Site-Specific Stable PSMA-Targeted Antibody–Drug Conjugate for the Treatment of Metastatic Castration-Resistant Prostate Cancer. Mol. Cancer Ther..

[127] Shen J., Pachynski R., Nordquist L.T., Adra N., Bilen M.A., Aggarwal R., Reichert Z., Schweizer M., Iravani A., Aung S. (2023). 1804P APEX-01: First-in-Human Phase I/II Study of ARX517 an Anti- Prostate-Specific Membrane Antigen (PSMA) Antibody-Drug Conjugate (ADC) in Patients (Pts) with Metastatic Castration-Resistant Prostate Cancer (mCRPC). Ann. Oncol..

[128] Hummel H.-D., Kufer P., Grüllich C., Seggewiss-Bernhardt R., Deschler-Baier B., Chatterjee M., Goebeler M.-E., Miller K., de Santis M., Loidl W. (2021). Pasotuxizumab, a BiTE^®^ Immune Therapy for Castration-Resistant Prostate Cancer: Phase I, Dose-Escalation Study Findings. Immunotherapy.

[129] Dorff T., Horvath L.G., Autio K., Bernard-Tessier A., Rettig M.B., Machiels J.-P., Bilen M.A., Lolkema M.P., Adra N., Rottey S. (2024). A Phase I Study of Acapatamab, a Half-Life Extended, PSMA-Targeting Bispecific T-Cell Engager for Metastatic Castration-Resistant Prostate Cancer. Clin. Cancer Res..

[130] Lim E.A., Schweizer M.T., Chi K.N., Aggarwal R., Agarwal N., Gulley J., Attiyeh E., Greger J., Wu S., Jaiprasart P. (2023). Phase 1 Study of Safety and Preliminary Clinical Activity of JNJ-63898081, a PSMA and CD3 Bispecific Antibody, for Metastatic Castration-Resistant Prostate Cancer. Clin. Genitourin. Cancer.

[131] Junghans R.P., Ma Q., Rathore R., Gomes E.M., Bais A.J., Lo A.S.Y., Abedi M., Davies R.A., Cabral H.J., Al-Homsi A.S. (2016). Phase I Trial of Anti-PSMA Designer CAR-T Cells in Prostate Cancer: Possible Role for Interacting Interleukin 2-T Cell Pharmacodynamics as a Determinant of Clinical Response. Prostate.

[132] Alzubi J., Dettmer-Monaco V., Kuehle J., Thorausch N., Seidl M., Taromi S., Schamel W., Zeiser R., Abken H., Cathomen T. (2020). PSMA-Directed CAR T Cells Combined with Low-Dose Docetaxel Treatment Induce Tumor Regression in a Prostate Cancer Xenograft Model. Mol. Ther.-Oncolytics.

[133] Narayan V., Barber-Rotenberg J.S., Jung I.-Y., Lacey S.F., Rech A.J., Davis M.M., Hwang W.-T., Lal P., Carpenter E.L., Maude S.L. (2022). PSMA-Targeting TGFβ-Insensitive Armored CAR T-Cells in Metastatic Castration Resistant Prostate Cancer: A Phase 1 Trial. Nat. Med..

[134] Uijen M.J.M., Derks Y.H.W., Merkx R.I.J., Schilham M.G.M., Roosen J., Privé B.M., van Lith S.a.M., van Herpen C.M.L., Gotthardt M., Heskamp S. (2021). PSMA Radioligand Therapy for Solid Tumors Other than Prostate Cancer: Background, Opportunities, Challenges, and First Clinical Reports. Eur. J. Nucl. Med. Mol. Imaging.

[135] Trautwein N.F., Brendlin A., Reischl G., Mattke M., Paulsen F., Loewenheim H., Zender L., la Fougère C., Dittmann H. (2024). PSMA-Guided Imaging and Therapy of Advanced Adenoid Cystic Carcinomas and Other Salivary Gland Carcinomas. Cancers.

[136] Wang J.H., Kiess A.P. (2023). PSMA-Targeted Therapy for Non-Prostate Cancers. Front. Oncol..

[137] Dall’ Armellina S., Aghakhanyan G., Rizzo A., Fanni S.C., Aringhieri G., Faggioni L., Cioni D., Neri E., Volterrani D., Morbelli S. (2025). PSMA-Targeted PET Imaging for Brain Metastases from Non-Prostatic Solid Tumors: A Systematic Review. Front. Oncol..

[138] de Galiza Barbosa F., Queiroz M.A., Nunes R.F., Costa L.B., Zaniboni E.C., Marin J.F.G., Cerri G.G., Buchpiguel C.A. (2020). Nonprostatic Diseases on PSMA PET Imaging: A Spectrum of Benign and Malignant Findings. Cancer Imaging.

